# An implementation manual for an interprofessional enhanced recovery after surgery protocol in cardiac surgery following international established frameworks

**DOI:** 10.3389/fcvm.2024.1392881

**Published:** 2024-07-22

**Authors:** M. E. Schmid, L. Dolata, H. König, S. Stock, S. G. R. Klotz, E. Girdauskas

**Affiliations:** ^1^ Department of Cardiothoracic Surgery, University Hospital Augsburg, Augsburg, Germany; ^2^ Department of Patient and Care Management, University Medical Center Hamburg-Eppendorf, Hamburg, Germany; ^3^ Department of Physiotherapy, University Medical Center Hamburg-Eppendorf, Hamburg, Germany

**Keywords:** implementation manual, implementation science, minimally invasive heart surgery, cardiac surgical procedures, enhanced recovery after surgery, perioperative care

## Abstract

**Introduction:**

Enhanced Recovery After Surgery (ERAS) protocols represent a paradigm shift in perioperative care, aim to optimize patient outcomes and accelerate recovery. This manual presents findings from implementing the INCREASE study, a bicentric prospective randomized controlled trial focusing on ERAS in minimally invasive heart valve surgery.

**Methods:**

Utilizing the Consolidated Framework for Implementation Research (CFIR) and the Template for Intervention Description and Replication (TIDieR), the study examined contextual factors, intervention components, and implementation strategies.

**Results:**

Key findings from the CFIR analysis revealed critical domains influencing implementation success. These included innovation characteristics, external and internal settings, and individual dynamics. The study showcased ERAS's adaptability to diverse healthcare systems, emphasizing its potential for successful integration across varying contexts. Furthermore, the importance of interprofessional collaboration emerged as a foundation of practical implementation, fostering teamwork, communication, and patient-centered care. Utilizing the TIDieR framework, this manual comprehensively describes ERAS intervention components, detailing preoperative counseling, intraoperative management, and postoperative care strategies. The manual underscored the importance of tailored, patient-centered approaches, highlighting the role of an academic ERAS nurse, early mobilization, and psychosomatic interventions in promoting optimal recovery outcomes.

**Discussion:**

In conclusion, the INCREASE study provided valuable insights for creating an implementation manual for ERAS in cardiac surgery, emphasizing adaptability, collaboration, and ongoing evaluation as key drivers of successful implementation. These findings have broad implications for improving patient care outcomes and advancing perioperative practices in cardiac surgery settings.

## 1 Introduction

Cardiac surgery has undergone significant advancements over the years, with innovations in techniques, technology, and perioperative care (1). In this era of modern medicine, the implementation of Enhanced Recovery After Surgery (ERAS) protocols in cardiac surgery has emerged as a significant paradigm shift in perioperative care. ERAS (cardiac) is a comprehensive, interprofessional approach optimizing patient care after major surgery. The ERAS (cardiac) protocol effectively reduces surgical stress, minimizes complications, and accelerates postoperative recovery. The primary goals are to treat cardiac disease and return patients to normal functional status as quickly as possible. This protocol has been proven to be highly effective in achieving these goals (2). Additionally, the components of ERAS in cardiac surgery (preoperative patient education, intraoperative management, postoperative optimization and early de-escalation) further demonstrated to shorten hospital stays, lower healthcare costs, and ultimately improve patient satisfaction without compromising safety (3–5). The positive outcomes of ERAS highlight the importance of spreading its adoption further, especially considering that its implementation in cardiac surgery is not yet widespread globally. This is because implementing ERAS requires significant organizational and cultural changes within healthcare systems.

ERAS was originally developed for colorectal surgery in the early 1990s. Since then, its principles have been successfully applied to various surgical specialties, including cardiac surgery, despite its complexity and the potential for significant postoperative complications. In the early 2000s, healthcare institutions and clinicians began adapting ERAS principles for cardiac surgery patients (6). In 2017, the ERAS Cardiac Society established and produced evidence-based guidelines for ERAS programs in cardiac surgery (7), which have been widely accepted and implemented. Nonetheless, the guidelines are designed to apply to cardiac surgery as a whole, without being tailored to a distinct surgical procedure. Hence, they are rather generic than suited for specific surgery methods. These guidelines cover various aspects of care and are a crucial resource for healthcare providers and institutions interested in adopting ERAS practices. The implementation of ERAS in cardiac surgery requires meticulous adjustment of crucial components, including preoperative optimization, intraoperative refinement, and post-operative streamlining.

Henceforth, when referencing ERAS, it pertains specifically to ERAS implemented in the context of cardiac surgery.

In order to address the significant gap in evidence regarding the implementation of ERAS in minimally invasive heart valve surgery, the INCREASE study (Interdisciplinary and Cross-Sectoral Perioperative Care Model in Cardiac Surgery: Implementation in the Setting of Minimally-Invasive Heart Valve Surgery) was initiated. This study is funded by the innovation fund of the Federal Joint Committee (01NVF19028) (8). It aims to thoroughly examine the effectiveness of an advanced and refined interprofessional ERAS protocol. The INCREASE study is a bi-centric prospective randomized controlled trial conducted at the University Hospital Augsburg and the University Medical Center Hamburg-Eppendorf. Following informed consent, all eligible patients were assigned randomly to one of two groups: standard care or the interprofessional and transsectoral ERAS program. This program involved collaboration among cardiac surgery, anesthesiology, physiotherapy, advanced nursing, social care services, and psychosomatic support. The ERAS program in the INCREASE study included preoperative patient education, which addressed physical activity, nutrition, expectation-focused intervention and psychological preparedness. Intraoperative management was adapted to facilitate early extubation, which enables postoperative mobilization within three hours after surgery. The program comprises active physiotherapy, opioid-sparing analgesia, and early discharge on the fourth to fifth post-operative day.

With this manuscript, we present a comprehensive manual for effectively implementing ERAS in cardiac surgery. By offering a structured roadmap, the manual aids healthcare providers, surgeons, and institutions in implementing ERAS according to the INCREASE protocol in other modern heart centers. This manual allows healthcare providers to confidently implement ERAS and achieve successful outcomes while adhering to the INCREASE protocol. Practical insights and step-by-step strategies for minimally invasive cardiac surgery are provided in this text. Throughout the implementation process, we have gained valuable experience and addressed specific challenges that may benefit other cardiac surgery departments planning to implement an ERAS protocol in minimally invasive valve surgery.

## 2 Methods

In this paper, we have combined the Template for Intervention Description and Replication (TIDieR) (9) with the 2022 updated Consolidated Framework for Implementation Research (CFIR) (10) to describe the implementation of ERAS as applied in the INCREASE study. The resulting manual can be used to replicate the intervention in other hospitals. TIDieR provides a detailed description of interventions, including what, why, how, where, and when an intervention is implemented (9). This level of detail is essential to ensure replicability and understanding of the core components of an intervention. Additionally, CFIR emphasizes the significance of context and implementation determinants, facilitating the identification and evaluation of the factors that impact the success or failure of an intervention within a particular setting. By using both frameworks, we can enhance the clarity and comprehensiveness of our reporting on the implementation of our ERAS program, which is particularly crucial for those seeking to understand or replicate it.

An expert panel of representatives from various professions collaboratively specified the TIDieR and CFIR frameworks for our ERAS program. The discussions occurred iteratively and involved professionals such as physiotherapists, nursing specialists, health scientists, medical professionals, and psychologists, all of whom were integral members of the ERAS program. The discussions were designed strategically to alternate between intra- and interprofessional development phases, resulting in a comprehensive approach. The panel was strengthened further by the inclusion of health scientists and health economists. Their expertise was instrumental in successfully developing and implementing the ERAS program. The interprofessional panel ensured a low hierarchy and inclusivity of perspectives beyond surgical expertise. The authors have successfully tailored TIDieR and CFIR to address program requirements and challenges, drawing on their extensive practical knowledge and experience. The interprofessional approach in this process ensures that the frameworks remain practical and relevant in various healthcare contexts. The following sections will provide detailed explanations of CFIR and TIDieR.

### 2.1 CFIR

The Consolidated Framework for Implementation Research (CFIR), developed by Damschroder et al. (11) in 2009 and updated in 2022 (10), is a widely recognized and comprehensive framework used to guide and understand the implementation of innovations, interventions, or programs in various settings, with a particular focus on healthcare and public health. CFIR offers a structured and systematic approach to studying and assessing the factors that influence the success of implementation efforts. Although originally developed for implementation research, the CFIR has been demonstrated to be valuable and helpful in guiding a practical implementation process (12). This framework is particularly well-suited for describing the ERAS program, as it reflects the complex nature of the INCREASE study and involves multiple components, stakeholders, and contextual factors. CFIR's constructs and domains are instrumental in identifying specific facilitators and barriers that affect the implementation process, allowing targeted interventions to address these challenges (10, 11). CFIR's interprofessional nature and consideration of patient perspectives make it especially suitable for understanding and implementing the ERAS program. It comprises five major domains: Intervention Characteristics, Outer Setting, Inner Setting, Characteristics of Individuals, and the Implementation Process. Within these domains, CFIR includes 39 specific constructs that guide the systematic assessment and understanding of factors influencing program or intervention implementation. These constructs cover various aspects, such as intervention complexity, external factors in the implementation environment, organizational and individual characteristics, and the dynamic implementation process.

### 2.2 TIDieR

The Template for Intervention Description and Replication (TIDieR) is a structured framework designed to enhance the comprehensive and transparent reporting of complex interventions in research and clinical practice (9). Developed by researchers and clinicians, TIDieR consists of a 12-item checklist that aids authors in providing precise and consistent descriptions of interventions. It includes essential details such as the intervention's name, rationale, materials, procedures, and delivery methods. By offering a standardized approach to documenting interventions, TIDieR promotes reproducibility, transparency in research, and the effective dissemination of knowledge (13). This contributes to a better understanding and implementation of interventions across various fields, including healthcare, education, and social sciences.

### 2.3 Ethics

The publication outlines the theory-based development of an implementation manual for an ERAS program in patients undergoing minimally invasive heart valve surgery. This project does not involve humans as study participants and as no data were collected, processed, or analyzed, no ethics approval was required. An ethics approval was obtained for the study investigating the effect of the ERAS program from the Ethics Committee of the Medical Association Hamburg, Germany (June 7th, 2021; reference number 2020-10276-BO-ff). However, the reporting of the effects is not part of this publication.

## 3 Results

In the following, we will first present the main topics of the CFIR. This is followed by the central factors of the TIDieR.

### 3.1 CFIR

**Figure 1:**
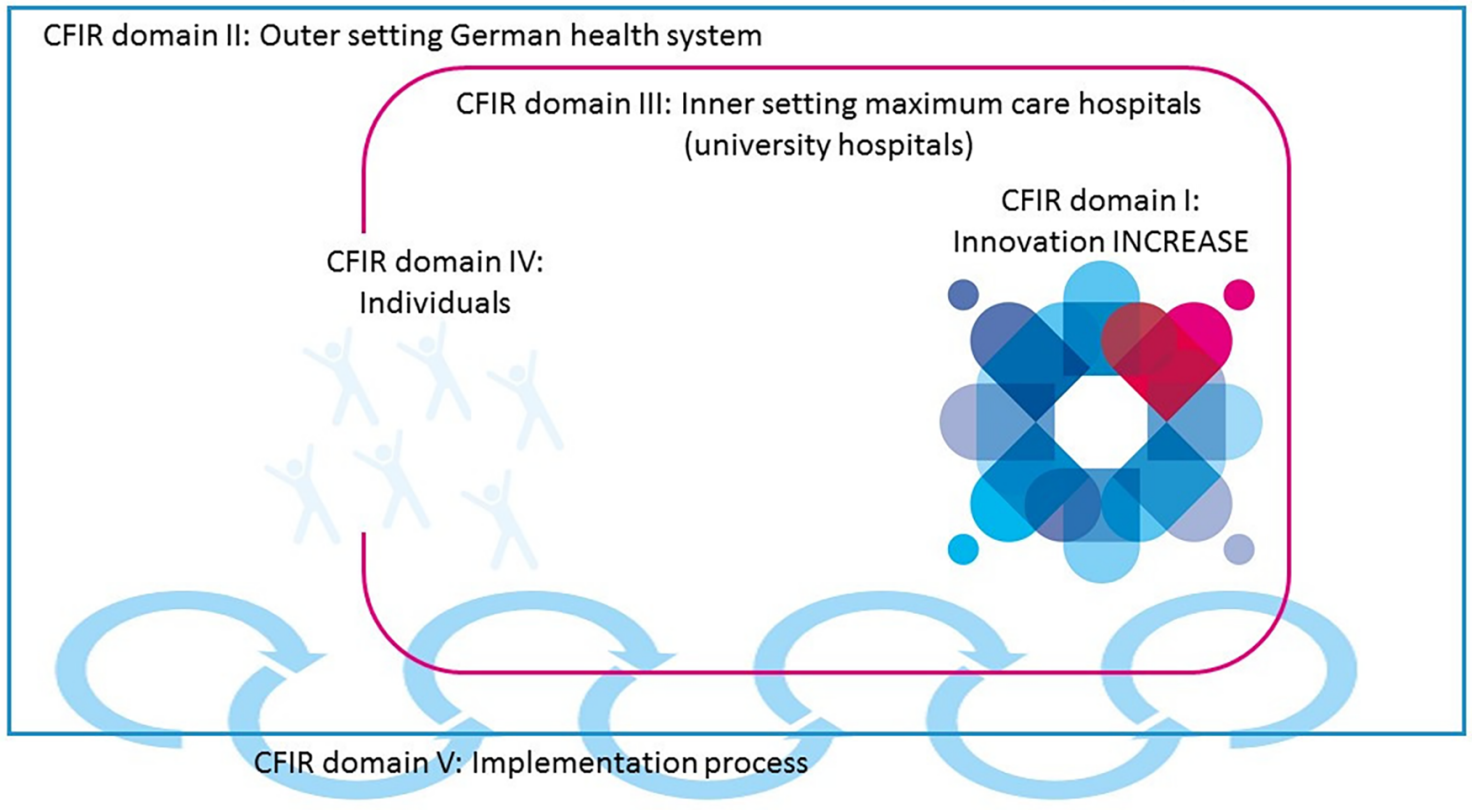
Major domains of the consolidated framework for implementation research (CFIR) for the INCREASE program. Figure shows the constellation of the five major domains of the CFIR (the INCREASE intervention, the inner setting maximum care hospitals, the outer setting German health system, the individuals involved, and the process by which the implementation of INCREASE is accomplished) and how the domains interact in complex ways with each other. Annotation: the logo used in this figure was designed especially for the INCREASE project representing the different professions working together with the patient.

Following the CFIR (Consolidated Framework Of Implementation Research) pathway, all factors, sites, and frameworks that influenced the implementation process for the INCREASE study were collected and recorded in a structured manner. Figure 1 shows the constellation and relationships of the different CFIR domains for the INCREASE program based on the presentation in the CFIR 2009 version (11). The detailed description of the CFIR domains can be found in the Supplementary Material 1.

I - Innovation domain: This category defines the innovations and deviations, improvements, and novelties from the current standard. For the INCREASE study, the ERAS guidelines of the ERAS Society were used and further developed. Additionally, this domain explains the origins of INCREASE, looking back to the journey of an interprofessional team from Hamburg to Brazil. The specially trained and educated nurse, an Advanced Practice Nurse (APN), plays a crucial role in the ERAS program. In addition, experiences and challenges from previous studies are taken into account. The benefits of ERAS have been demonstrated in several studies. Compared to standard cardiac surgery, ERAS has been shown to reduce the incidence of post-operative atrial fibrillation, shorten intubation times, and reduce the length of stay in the intensive care unit and hospital stay. Additionally, ERAS has the potential to reduce pain intensity, decrease the need for opioids, improve early postoperative mobility, expedite oral feeding recovery, and reduce costs (14).

II - Outer Setting domain: To ensure transferability to other clinical settings in different countries, it is necessary to explain the external circumstances, including the German healthcare system and hospital structures in Germany. In addition, the political, legal, and financial context of the study are described. Challenges that arose during the implementation of our ERAS program in the frame of the INCRASE study include the consequences of the COVID-19 pandemic, which led to a reduction of elective surgeries (15, 16). Another noteworthy challenge was the development of patient empowerment (including the relatives of patients) in German healthcare facilities. Nonetheless, patients were actively involved in the care process as part of the ERAS program. They were encouraged to act autonomously and to participate actively in treatment planning, which had a major impact on the tightly scheduled postoperative course of treatment.

III - Inner Setting domain: This section examines the internal clinical structures, including the unique relationships and networks formed and the formal and informal connections necessary to innovate and implement the INCREASE trial in two different university hospitals. As the study protocol includes cardiac surgical procedures, it is advantageous to have an operating room designed explicitly for the ERAS program. Patients enrolled in the INCREASE study require a low-care postoperative environment outside of the Intensive Care Unit (ICU), and their monitoring is mainly limited to an overnight stay. Therefore, the Post Anaesthetic Care Unit (PACU) or Intermediate Care Unit (IMC) helps reduce the ICU burden. This concept is beneficial for the patients and is advantageous when considering the limited human resources and exponentially high costs of an ICU (17). Interprofessional networks and regular weekly interprofessional meetings were established to implement and evaluate the ERAS process. Intraprofessional meetings were also introduced to implement the new model, to internalize the newly developed standards as a team, and to train and empower the clinical staff in its use. The ERAS team was provided with hospital facilities to conduct these meetings, training, and consultations with patients and staff.

IV - Individuals domain: The Individuals Domain describes the dynamic interaction between individuals and organizations in the ERAS program. It defines and records the Need, Capability, Opportunity, and Motivation of individual high-level, mid-level, and opinion leaders of the University Hospitals of Augsburg and Hamburg-Eppendorf. The table records all interventions and measures, including evidence-based care, work requirements, compliance with and implementation of Standard Operating Procedures (SOPs), examples of ERAS implementation, knowledge of ERAS, and competencies in the relevant steps for each profession. It includes beliefs and strategies for implementing ERAS and transferring it to patients.

V - Implementation Process domain: This domain delineates the pertinent activities and strategies employed to operationalize the innovation. In addition to these activities and strategies, a number of prerequisites, particularly for the various professions but also cross-sectoral, can be found in the TIDieR. These prerequisites must be implemented prior to the commencement of the INCREASE program.

Using the CFIR helps to identify improvements in implementation strategies for future practice transformations in other clinical settings leading to enhanced implementation effectiveness. Furthermore, we have identified challenges and characteristics that require special attention, such as the physical space needed to carry out preoperative consultations and patient training, as well as regular meetings and exchanges in the interprofessional team.

### 3.2 TIDieR

All measures and interventions performed by each profession involved in the patient's care, including cardiac surgery, anesthesia, nursing, physiotherapy, and psychosomatics were documented in a tabular list. These components are listed as items. Perioperative care typically includes anesthesia and individualized hemodynamic therapy, care during cardiac surgery, individualized nursing, and physiotherapy focusing on goals and self-efficacy, including early mobilization. Additionally, a psychosomatic intervention is provided to address expectations. Table 1 gives an overview of the items of the TIDieR. The detailed description of the TIDieR domains can be found in the Supplementary Material 2.

**Table 1:** Overview of the items described in the TIDieR checklist in detail.

Professions involved in the INCREASE Intervention
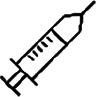 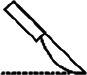 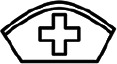 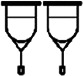 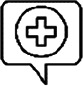				
Anesthesia Cardiac surgery Nursing Physiotherapy psychosomatics				
Why	Rationale, theory, or goal behind the elements of the intervention 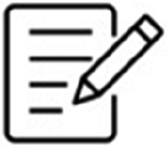 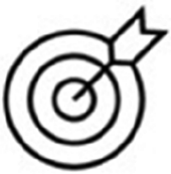			
What	Materials and procedures used in the intervention 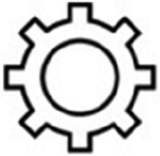			
Who provided	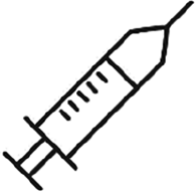 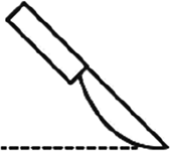 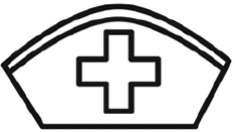 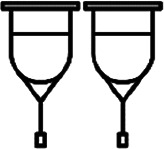 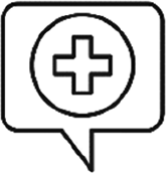			
How	Modes of delivery of the elements of the intervention 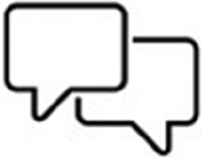 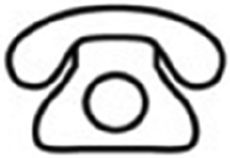			
Where	Locations of the elements of the intervention 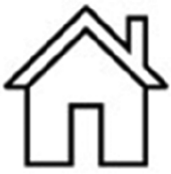			
When, and how much	Period of time, sessions, schedule, duration, intensity, and/or dose of the elements of the intervention 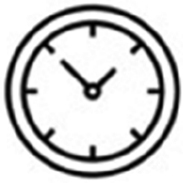			
Tailoring	Personalization of the intervention 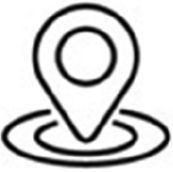			

Annotation: vector graphics from Kinggod and Stockgiu at vecteezy.com.

Item 1 “Name” provides a name or brief description of an intervention to facilitate identification and linkage with other reports. Preoperative counseling and training by professionals aim to reduce patient anxiety and improve compliance (7). By training and implementing standardized interventions, ERAS patients become active participants in their recovery process to promote faster healing. The ERAS nurse is essential in this regard, working with the interprofessional team to increase patient self-efficacy and health literacy.

Item 2 “Why” describes the rationale, theories, or objectives underlying an intervention or a complex intervention's components. This can help to distinguish which elements are essential, optional, or incidental. For instance, interventions that are used but are optional to the effectiveness of the intervention do not need to be reported. All professions should adhere to the ERAS Society guidelines and institution-specific SOPs. For example, in anesthesia, intraoperative anesthetic management aims to maintain a stable fluid balance, stabilize hemodynamic conditions, and relieve postoperative pain, nausea, and delirium. Postoperative management in the PACU aims to facilitate early mental and physical recovery.

Item 3 “What” includes any information or physical material used in the interventions, including materials distributed to study participants or used during treatment or clinicians education. This includes specific items such as nutrition and hydration management, worksheets, educational materials, and a diary developed to individualize goal setting with patients within the ERAS program.

Item 4 “Procedure” describes the procedures, activities, and processes used including preparatory and supportive activities. For example, in nursing, the ERAS nurse provides personalized education and counseling to patients and their families, tailored to their individual needs and resources during the various phases of the perioperative process. Motivational interviewing is an integral part of the interprofessional approach.

Item 5 “Who Provided” documents the individuals who intervened, considering the expertise of the professionals, their background, and any specialized interprofessional team training. The perisurgical anesthetic care was provided by either an anesthesiologist experienced in cardiac anesthesiology and cardiac intensive care medicine. The physiotherapeutic intervention, for example, was provided by a physiotherapists with at least an EQR 6 entry-level qualification.

Item 6 “How” describes how the intervention was performed, including whether it was delivered as an individual or group intervention. The interventions in the ERAS program, including patient counseling and training, were delivered in person. Although the involvement of relatives was explicitly desired, another aspect of ERAS is actively involving them in the therapy.

Item 7 “Where” records and describes the characteristics of the sites where the interventions were carried out, including any necessary infrastructure or relevant special features. The ERAS interventions and procedures took place in different hospital wards, so the locations may vary. A separate room with chairs and a table would suit the preoperative consultation, offering privacy and a quiet atmosphere. The first postoperative meetings were held in the IMC or ICU, while subsequent meetings were held in the peripheral wards, in the corridor, in the stairwell, or on the hospital grounds. No particular infrastructure is required, and the intervention can be adapted according to the patient-centered goals and the environment. Conditions for conducting the patient follow-up via telephone were quietness and privacy in the clinical area.

Item 8 “When and How Much” describes the number of times the intervention was delivered and over what period of time including the number of sessions, their schedule, and their duration, intensity or dose. All professions include at least one pre-surgical intervention and several post-surgical interventions.

Item 9 “Tailoring” describes that all interventions in the different professions are individualized depending on the patient´s individual goals and the bio-psycho-social conditions.

## 4 Discussion

The main objective of this paper was to provide a detailed manual for implementing the ERAS program as applied in the INCREASE study in cardiac surgery in other institutions willing to implement ERAS. By applying CFIR, we identified numerous contextual factors and components critical for the successful implementation of this ERAS program. We must acknowledge the barriers and challenges we encountered during the implementation process. Furthermore, it is crucial for stakeholders and individuals to understand the benefits. Effective communication among professionals is a critical element in the implementation process.

The TIDieR of our ERAS program includes detailed descriptions for each profession involved, such as anesthesia, cardiac surgery, nursing, physiotherapy, and psychosomatics. We have identified crucial aspects that the ERAS program must encompass. These include the presence of an academic ERAS nurse, a preoperative interprofessional outpatient educational session, early extubation, de-escalation, and intensive physiotherapy.

To understand the meaning of these outcomes, it is crucial to consider the context (CFIR definitions) in which we implemented the ERAS program, two university hospitals in Germany. Different countries and settings present unique circumstances, particularly concerning the outer setting, which require careful consideration (18). Thus, we recommend adapting the CFIR to meet the distinct healthcare setting and country before introducing our ERAS program.

For each profession, TIDieR presents a pragmatic framework. The described framework provides an overview and a possible structure for implementing ERAS in cardiac surgery. Nevertheless, it may be necessary to adapt the details of each intervention according to the local infrastructure and specific circumstances. The following discussion is paramount in understanding the complexity of implementing our ERAS program.

### 4.1 Adaption in different hospitals and countries

Through the implementation of our ERAS program at two locations - Augsburg and Hamburg - we have found that despite its complexity, the program is highly adaptable and reproducible. Nevertheless, the following fundamental components should be strongly considered. However, each hospital can incorporate individualized details to adapt to its needs. For instance, the academic ERAS nurse is essential for successfully implementing our ERAS program, preoperative patient education, and intensive physiotherapy. The practical execution of these components must be tailored to suit individual circumstances. The ERAS nurse, with their academic specification, is the initial point of contact and a permanent component for the patient throughout the entire perioperative process. The role of the ERAS nurse was developed and adapted specifically for the standardized ERAS concept. The ERAS nurse is not a study nurse; rather, their focus is on the perioperative therapeutic treatment of patients in the interprofessional team that is responsible for implementing the ERAS concept. The ERAS nurse must be able to identify deviations from the procedure and implement appropriate adjustments based on evidence.

During the preoperative patient session, the individual appointments were arranged differently. The psychosomatic session was scheduled at the end, while the other sessions were adjusted and occasionally adapted to the clinical needs. Physicians faced challenges in adhering to schedules, given their multitask responsibilities. Additionally, in Hamburg the patients were transferred to the PACU after surgery, whereas they were transferred to the IMC unit in Augsburg. In the transition phase from the INCREASE trial to the standard care (i.e., without governmental or hospital funding), the psychosomatic session during the preoperative patient education visit was abandoned for most patients in Hamburg. However, in Augsburg it was still possible to cover the expectation-focused intervention due to the different availability of personal resources. Despite our belief that psychosomatic support is crucial for patients and their relatives, it may require further adjustments when considering the time constraints and financial resources. However, our long-term goal is to reintroduce psychosomatic support in Hamburg once health insurance companies can cover it as patients experienced the expectation-focused intervention as helpful (19).

This variability in specific ERAS details demonstrates the flexibility of our program. There is an extensive toolbox of ERAS elements to adapt the program to the specific requirements of each hospital or healthcare system. Local factors like healthcare regulations, patient demographics, and resources may necessitate tailored modifications.

### 4.2 Interprofessional collaboration and communication

Efficient interprofessional and intersectoral communication was a critical factor for the success of the ERAS program. This communication not only integrated all stakeholders but also fostered a strong network and team spirit, which aligns with the literature about interprofessional communication and team building (20). Interprofessional coordination and inclusion of the patient's goals are crucial for the implementation and success of the standardized and time-limited process. Its positive impact can be seen throughout various healthcare processes, both within and outside the hospital, benefiting the program and patients.

An ERAS nurse organizes and coordinates regular meetings with the patients and the interprofessional team. In addition, the ERAS nurse keeps close contact with the patients, their families, and the interprofessional team to design and communicate the individual therapy plan and the current recovery status. The ERAS nurse has a key position with all organizational and professional threads coming together, accompanying patients and colleagues, and enabling patient participation. Requirements for this responsible task is nursing expert knowledge and an academic qualification (21).

The focus on extensive network building and teamwork improved perioperative care and facilitated more intense collaboration with the referring clinics, external physicians, and rehabilitation facilities. This increased collaboration led to smoother patient transitions through different healthcare stages, promoting healthcare continuity and patients' trust.

The interprofessional ward rounds, meetings, and feedback talks were crucial to enhancing teamwork and collaboration among healthcare professionals at various levels. These interactions provided a platform for healthcare providers to exchange critical insights, strategize patient care coordination, and manage all arising challenges. The shared knowledge and expertise reinforced the spirit of teamwork and promoted a culture of continuous medical education in which healthcare professionals could learn from each other's expertise. This learning effect is described in the literature as interprofessional education (22, 23). The existing body of evidence supports these benefits of interprofessional ward rounds (24–27) and interprofessional education (23, 28, 29).

The achievement of our ERAS program in encouraging interprofessional collaboration and communication underscores the critical role of teamwork in healthcare. By breaking established barriers, creating a sense of shared responsibility, and ensuring effective communication, healthcare providers can improve patient outcomes and revolutionize how healthcare is delivered, both within the hospital and throughout the healthcare ecosystem (30, 31).

During the development of this manual, collaboration with professionals from various disciplines and with different responsibilities during the implementation process was crucial. Their pooled expertise and experiences enhanced the development of a manual that accounts for a broad range of perspectives and practical considerations, ultimately improving its effectiveness and applicability.

### 4.3 Monitoring and evaluation

Although CFIR is a complex framework, it has been shown to be helpful in ensuring that all critical details are sufficiently covered. The ERAS program, being heavily influenced by multiple factors, benefits greatly from the CFIRs as an all-inclusive tool for capturing many aspects of complex processes that might be important. Working extensively with CFIR provided us with new insights that are potentially relevant for the future of our ERAS program after the initial implementation. There are still challenges to be faced, and it is still a work in progress. Therefore, working with CFIR to implement a new healthcare model can be advantageous as it facilitates the preparation and identification of the pertinent aspects of the subject matter (32).

The INCREASE study started in 2020 and we have gained short- to mid-term experience with the implementation and the post-implementation. However, there are no long-term insights to report at this time. Gaining long-term insights and conducting ongoing monitoring and evaluation is crucial to understanding the sustained impact of the ERAS program. Although we acknowledge the absence of long-term insights into ERAŚ impact, it is important to emphasize the significance of this gap and the potential value that future research addressing it can bring.

### 4.4 Practical implications for implementing ERAS as applied in INCREASE in other hospitals

**Figure 2:**
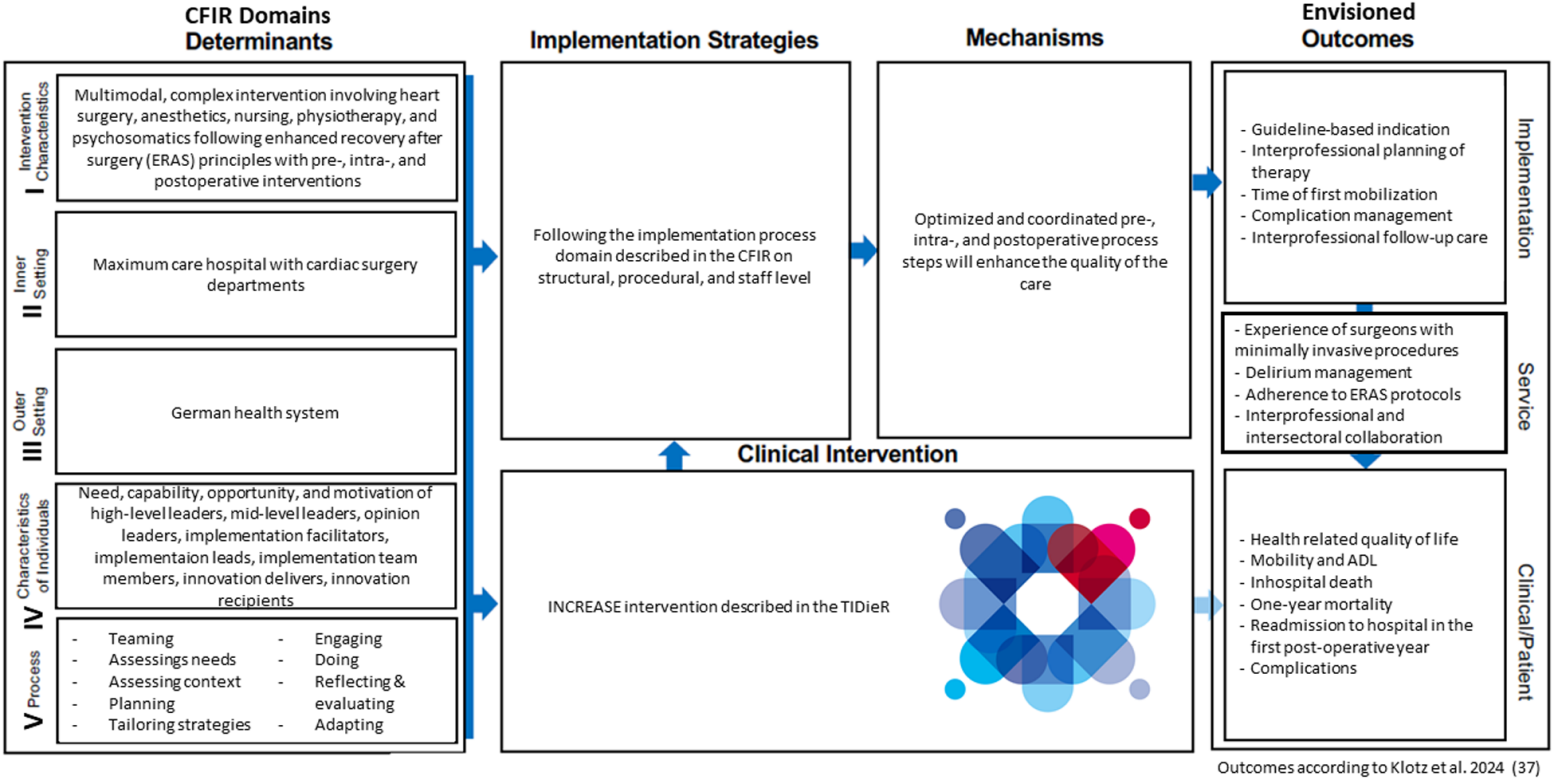
Implementation process of INCREASE following the steps of the implementation research logic model (34). Adapted with permission from Smith et al. (36), licensed under CC BY + CC0, Source: https://figshare.com/articles/journal_contribution/Additional_file_2_of_The_Implementation_Research_Logic_Model_a_method_for.

Table 2 provides valuable practical implications for the implementation of ERAS as used in INCREASE in other hospitals. Furthermore, the Implementation Research Logic Model (36), including envisioned outcomes according to a newly developed core outcome set could be helpful in the implementation process of INCREASE. We have displayed the implementation process of INCREASE using the CFIR within the Implementation Research Logic Model in Figure 2. The envisioned outcomes described in the figure are based on our previous work in which we determined a core outcome set for ERAS in minimally invasive cardiac surgery.^1^

**Table 2:** Practical implications for implementing ERAS as applied in INCREASE in other hospitals.

Adaptability to different healthcare systems & resource flexibility 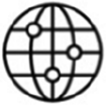 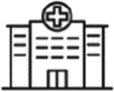	The adaptability and flexibility of the ERAS program as applied in INCREASE is a major finding from its implementation in Augsburg and Hamburg. This suggests that the program can be effectively introduced into healthcare systems with varying structures, funding models, and regulations and it is adaptable to settings with different resource levels. For instance, the program proved successful in a German university hospital setting. However, its basic principles can be adapted to meet the needs of public or private healthcare facilities elsewhere. Moreover, in hospitals or clinics with limited resources, the program's core elements can still be integrated. Although some creativity and resource allocation adjustments may be needed, the program can be implemented successfully in resource-rich and resource-constrained environments.
Local regulatory compliance 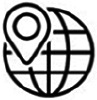	Compliance with local healthcare regulations and guidelines is of paramount importance. Future implementations of ERAS must conform to the specific regulations of the host country or region. This may require close cooperation with healthcare authorities to ensure compliance with local laws and standards in certain circumstances.
Patient-centered modifications 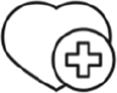 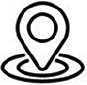	Patient-centered care is a core principle of the ERAS program. Patient education, counseling, and postoperative care should be tailored to the specific needs and expectations of patients in their area by healthcare professionals working in different contexts. This requires overcoming language barriers, respecting cultural preferences, and developing tailored care plans.
Interprofessional collaboration 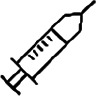 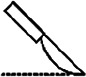 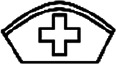 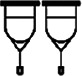 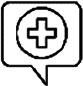	Effective interprofessional and intersectoral communication played a vital role in the success of our ERAS program in Augsburg and Hamburg. We suggest that other hospitals prioritize establishing solid networks and fostering teamwork among healthcare providers. Moreover, it is highly advantageous to regularly engage with hospitals that have already implemented ERAS and visit them for firsthand insights.
Monitoring and evaluation protocols 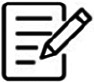	The establishment of strong monitoring and evaluation procedures is crucial for future implementations. These protocols enable healthcare providers to continuously assess the program's impact and identify areas requiring adjustments over time. Regular performance evaluations and outcome assessments guarantee long-term success. Examples for such evaluation tools can be Plan-Do-Study-Act cycles (33), Patient-reported Outcome Measures (34), or the definition of key performance indicators (35).

Annotation: vector graphics from Kinggod and Stockgiu at vecteezy.com.

### 4.5 Strengths

This study has several notable strengths that contribute significantly to its importance in implementing ERAS protocols in cardiac surgery. First, using two well-established frameworks, CFIR and TIDieR, adds rigor and comprehensiveness to our study (12, 18, 37). The synergy between these frameworks is beneficial because CFIR helps identify contextual factors critical to implementation success, while TIDieR ensures a detailed and standardized description of intervention components (18). This combination not only enriches the depth of our analysis but also facilitates a more holistic understanding of the implementation process.

Second, our work represents a pioneering effort to provide a comprehensive, step-by-step implementation guide for implementing our ERAS program. An extensive literature review identified a lack of implementation guidelines that provide all the necessary information in a single work. Our manual fills this gap and serves as a practical, accessible resource for healthcare professionals seeking to implement ERAS according to the INCREASE protocol in their respective settings (32).

Third, our implementation manual adheres to the recommendations by Proctor et al. (38), which enhance the reporting of implementation strategies. By following their guidelines during development, our study strengthens the transparency and completeness of our approach, aligning with best practices in the field.

In addition, our manual's step-by-step nature streamlines the translation of research into practice. This accelerates the implementation process and provides a clear roadmap for healthcare professionals. Unlike traditional research-to-practice timelines, which can span years, our manual may enable rapid integration into clinical practice, promoting timely adoption of evidence-based practices. Morris et al. pointed out that the time lag in developing health interventions is 17 years (24).

### 4.6 Limitations

A notable limitation of our study is the lack of a formal evaluation of the implementation process. While we carefully evaluated the outcomes of the INCREASE study, we did not specifically evaluate how well the implementation manual was executed. We acknowledge that the development of the manual was conducted within an expert group in an unstructured manner, without the inclusion of external experts or a Delphi panel, which could have provided diverse perspectives and refined the methodology. This limitation suggests the need for future research to incorporate a more structured approach involving a broader range of experts to increase the robustness and generalizability of the implementation manual.

While our study notes the limitation of an unstructured development process, Oyebode et al. (39) emphasize the valuable role of expert opinions in complementing empirical evidence. This recognition underscores the importance of incorporating diverse expert perspectives to refine methodologies and enrich evidence-based guidelines, which aligns with the call for more structured approaches in future research.

Another limitation is the need for more objective data on the success of the INCREASE study and its ERAS program. The absence of INCREASE results in our study limits our ability to determine the success of implementation conclusively. At this point, we can only speculate about the effectiveness of the manual based on its principles and the preliminary results observed. The upcoming INCREASE results, expected in 2024, will provide essential data to assess the program's impact and address this limitation objectively. Until then, any statements about implementation success should be viewed cautiously, emphasizing the ongoing nature of our understanding and evaluation of our ERAS program.

### 4.7 Implications for research

A key area for future research is to conduct comprehensive and longitudinal evaluations to measure the lasting impact of implementation guidelines in healthcare settings. The use of mixed-methods approaches that include qualitative assessments of stakeholder experiences alongside quantitative measures to assess guideline adherence and outcomes over time may provide a more nuanced understanding.

Moreover, further research on the implementation of ERAS protocols should focus on patient-centered outcomes and experiences. Understanding how patients perceive and engage with implemented guidelines will be critical to refining guidelines to meet better the diverse needs and expectations of the patient population.

Integrating additional implementation science models beyond CFIR and TIDieR deserves attention in future research. Exploring the use of alternative frameworks and models could enrich our understanding of the various factors that influence successful implementation and contribute to a more comprehensive knowledge base for the development of future guidelines.

Furthermore, research efforts should focus on examining the long-term effects of interprofessional collaboration on the sustainability of implemented programs. A specific focus on how different healthcare disciplines continue to work together over time can provide valuable insights into the challenges faced and the successful strategies employed to maintain effective teamwork beyond the initial implementation phase.

## 5 Conclusion

In summary, the experiences from the University Hospitals of Augsburg and Hamburg, as presented in this paper, offer valuable insights for implementing ERAS as applied in the INCREASE study in other hospitals. The program's adaptability to different healthcare systems, resource flexibility, local regulatory compliance, patient-centered modifications, interprofessional collaboration, and ongoing monitoring and evaluation protocols are key takeaways for future implementations. As we move forward, we anticipate that these insights will contribute to the continued success of our ERAS program, ultimately leading to improved patient care in cardiac surgery.

## Data Availability

The original contributions presented in the study are included in the article/**Supplementary Material**, further inquiries can be directed to the corresponding author.

## Footnotes

^1^
Klotz SGR, Begerow A, Girdauskas E. Development of a core outcome set for Enhanced Recovery After Surgery in minimally invasive cardiac surgery. Results of an interprofessional Delphi consensus study. (2024).
