# Supplementary material for: An implementation manual for an interprofessional enhanced recovery after surgery protocol in cardiac surgery following international established frameworks
Source: Front Cardiovasc Med. 2024 Jul 22;11:1392881. doi: 10.3389/fcvm.2024.1392881 (PMC11298346; doi:10.3389/fcvm.2024.1392881)
Supplement: Supplementary file 1 [file Table1.pdf]

Descprition of the INCREASE Intervention Using the Template for Intervention Description and Replication (TIDieR)

	Anaesthesia	Cardiac Surgery	Nursing	Physiotherapy	Psychosomatics
**BRIEF NAME**	Perisurgical anesthetic care with individualized hemodynamic therapy focused on early postsurgery recovery	Perisurgery healthcare in cardiac surgery	Individualized nursing in pre- and postsurgery care process	Pre- and post-surgery, individualized, goal-oriented, and self-efficacy-based physiotherapy with very early mobilization	Pre- and post-surgery expectation-focused psychosomatic intervention
**WHY**	The rationale behind the anesthetic management is to educate the patient pre-surgery about the planned anesthetic action to reduce fear and to increase compliance in the postsurgery period. The intra-surgery anesthetic management aims to maintain a stable fluid balance, stabilize hemodynamic conditions, and reduce postsurgery pain, nausea, and delirium. The postsurgery management in the PACU aims to support early mental and physical recovery. The main goal is to reduce the	According to the Guidelines of the ERACS (enhanced recovery after cardiac surgery) society (1), fast and complete recovery by providing evidence-based and standardized multidisciplinary perisurgical care.	The ERAS Society highly recommends an ERAS nurse (2). The pre-operative intervention aims to educate, advise the patients, and familiarise them with the ERAS protocol (3-5). Furthermore, the ERAS nurse tries to encourage the patients to achieve their goals after surgery (6). During the hospital stay, the ERAS nurse is the main coordinator and contact person for the patients and their relatives (4). The ERAS nurse coordinates and organizes all therapies the patients need. In addition, constant evaluation of the care processes as well as presentation and discussion of the results with the interprofessional team to improve patient care is part of the taks of the ERAS nurse (4, 7).	The intervention aims at decreasing the amount of time patients spend in hospital (8) and thereby reducing the costs for society (9, 10). Well-educated and highly involved patients are able to contribute maximally to their own recovery and, in doing so, will speed it up (11).<br>The goal of the pre-surgery intervention is to ensure patients best possible activity and fitness level going into surgery (12, 13) and to prepare the patients for the procedure and the hospital stay (14). Furthermore, fear should be diminished by letting the physiotherapists explain the proceedings and what to expect following the operation (15).<br>During the pre-surgery intervention, the physiotherapists emphasize the important role patients have in their preparation for and their recovery after the surgery, strengthening the patients´ self-efficacy and empowerment (16-18).<br>The rationale behind the individualized goal orientation is that if patients have to set their own goals, it will heighten their involvement and strengthen their resolve to adhere to their individual program (14, 19, 20). The individualized goals can also	Patients with negative illness perceptions show a lower physical quality of life and higher stages of heart failure according to the NYHA classification than patients with positive illness perceptions (31). Psychological support is one of the missing needs expressed by patients with cardiac diseases (32). Within the PSY-HEART trial, different psychological interventions for patients undergoing heart surgery were compared (33). Within this trial, the expectation-focused intervention improved the long-term outcomes such as mental quality of life, higher fitness for work, and lower disability scores significantly positively compared to an intervention focused on emotional support only or standard medical care.

	physical and mental trauma of the surgery as low as possible (1).			strengthen the patients' self-efficacy and improve clinical outcomes (21, 22).<br>The very early mobilization is an integral part of every ERAS program, having multiple effects like prevention of postoperative complications, higher functional outcome at hospital discharge, and reduction of length of hospital stay (23-26) Moreover, patients mobilizing themselves early and dressing up in their own clothes overcome the culture of bed rest and the inactive patient role (27-30).	
Diary	During the preoperative interprofessional outpatient educational session, patients receive a printed diary. The diary consists of two parts: the first part contains general information on nutrition, a checklist for the hospital stay, about the admission day, the day of the surgery, and the first period after the hospital stay, as well as specific information from the different departments (nursing, physiotherapy, and psychosomatic). The second part contains worksheets, which can be edited before their hospital stay, and diary pages to fill in during their stay.				
**WHAT:**					
**Materials**	The anesthetic care follows the Guidelines of the ERACS Society (1) and the in-house SOP for the intra- and postsurgery management of ERACS patients (34). No additional material is used. The key elements of anesthetic care in ERACS are 1) pre-surgical education about the anesthetic procedures 2) intrasurgical advanced monitoring, and medication using narcotic drugs with	Every element of cardiac surgical care is documented in institution specific Standardized Operating Procedures (SOP), which can be assessed online via the intranet. The surgical care follows the Guidelines of the ERACS Society (1). No additional material is used.	The patient diary contains information on how to prepare for the perioperative process, information on how to behave in the event of complications, the contact details of the ERAS nurse. In addition, patients and relatives receive information and tasks to do at home on nutrition and drinking quantity management.	Educational material and worksheets as part of the patient diary. The educational material for physiotherapy contains information about the recommendations for physical activity from the World Health Organization and how to integrate physical activity into daily life as preparation for the surgery but also for maintaining an active lifestyle after the surgery. The worksheets are designed for individual goal setting in the short-, middle- and long-term and to reflect on the physical activity during the pre- and postsurgical phase.	Several worksheets were introduced as part of the patient diary focusing on expectations. The patients completed the worksheets with the psychologist during the first face-to-face consultation a few weeks before the surgery and could complement them at home. The material included a page focusing on positive activities after the surgery and setting goals concerning the most important behaviors for cardiac health. The patients received an overview of the typical progress after the surgery and noted the developed strategies to deal with side effects. As homework, the patients should write a letter to themselves, which they received six weeks after the surgery. The material was based on the expectation-focused intervention described in the EXPECT manual from the PSY-HEART trial (35, 36).

	short half-life, multimodal pain management, and prophylaxis of nausea, vomiting, and delirium, and 3) post-surgical promotion of the early physical in mental recovery in the PACU.				
Preoperative interprofessional outpatient educational session	The preoperative interprofessional outpatient educational session is appointed two to six weeks before the surgery. It is held in the form of face-to-face conversations. The session should be in a quiet room that provides privacy and a calm atmosphere. The room should be equipped with chairs and a table.<br>The following professions each have a conversation with the patients (the approximate time is listed in parentheses): cardiac surgery (30 min), anesthesia (15 min), nursing (30 min), physiotherapy (45 min), and psychosomatic (60 min). We recommend holding the cardiac surgery clarification at the beginning of the session and the psychosomatic education at the end of the session. Furthermore, we recommend that patients bring their relatives to the session.<br>Moreover, the patients should bring their diary to this session (which they received in advance), as there are several worksheets from nursing, physiotherapy, and psychosomatic to work on during and after the session. The overall goal of the session is to give patients the opportunity to prepare themselves physically, mentally , and nutritional in the best way possible and to clear open questions.				
**Procedure**	The anesthetic care follows the inhouse SOP for the intra- and postsurgery management of ERACS patients (34). No additional material is used.	In the preoperative course, the cardiac surgeon educates the patient about the individual surgical procedure. Intraoperatively, the surgeon focuses on reduction of surgical trauma, i.e. the use of minimally invasive surgical access routes. During the postoperative course, key factors of surgical care are early deescalation (e.g. of drainage and catheters), promoting physical and mental recovery,	The ERAS nurse trains and advises the patients and relatives individually, specifically according to their needs and resources during the various phases of the perisurgery process. Content includes nutrition and drinking quantity management, the delegation of tasks for independent recording, such as daily weighing, evaluation, and therapy design in an interprofessional team with the patients. Motivational interviewing is another crucial part of the concept. Topics such as pain and	The different procedures are subject to the individual goals of the patients and hence vary across patients. In general, they include education, very early mobilization after the surgery, and further physiotherapy sessions. Typical topics during education are goal setting, self-management, physical activity, and a physically active lifestyle as preparation for the surgery and after rehabilitation. In addition, the entries on the worksheets and in the patient diary are reflected together with the patient. Examples ofthe content of the further physiotherapy sessions are elaboration of physical activities like walking, stair climbing, cycling, activities of daily living, respiratory physiotherapy, or exercises in different positions (e.g., lying, sitting, standing).	Within the first face-to-face consultation (two to three weeks before the surgery), the subjective theory of disease and the expectations towards the surgery are assessed. The worksheets are introduced and completed by the patient supported by the psychologist. Topics like mental preparation for the surgery and dealing with anxiety, if needed, are addressed. An imagination exercise is conducted, and questions based on the SCID interview take place to evaluate psychological comorbidities. Between the first and the second consultation, the patient writes the letter to themselves and complements the worksheets at home. If there is any need, telephone

		patient empowerment and adequate, opioid-sparing analgesia to enable intensive physiotherapy.	wound management, daily therapy planning with a focus on promoting self-management skills, and mobilization are evaluated daily through visits.<br>Working with the diary and handouts simplifies and supports the implementation of individual client-centric measures in the standardized process.		consultations with the psychologist are possible in between the consultations.<br>The second consultation takes place one day prior to surgery. Within this consultation, the expectations are evaluated. Furthermore, support and space for reflection are given depending on the needs of the patients.<br>During the days after surgery, strategies are reviewed, and expectations are reflected according to the individual needs of the patients.<br>The last consultation takes place four to six weeks after surgery via telephone. The content is individual, depending on the patient´s needs. At the same time, the patient´s letter will be sent to their home address.<br>If there is any further need within the first months after surgery, the patient can contact the psychologist for further telephone consultation.
	Postsurgical: daily interprofessional rounds in the morning with the five professions participating; they take place at the IMC/PACU/ICU, and the peripheral ward; the interprofessional team, together with the patient, and, if applicable, with affiliated persons or relatives, reflects about the day before and discusses the plans for the current day.				
**WHO PROVIDED**	Anesthesiologist with experience in cardiac anesthesiology, and cardiac intensive care medicine.	The preoperative education is preferably performed by a consulting cardiac surgeon, alternatively by an experienced intern with profound knowledge about the planned surgical procedure. Intraoperatively,	The ERAS nurse is a trained nursing specialist with a master's degree.	Physiotherapists, preferably having at least entry-level EQR 6, provided the physiotherapeutic intervention. There is no specific training beyond the regular qualification needed; however, the therapists should familiarize themselves with the ERAS concept, the process of goal setting, and the competencies needed for effective education, e.g., skills in Motivational Interviewing (37). Before the therapists	Psychologists or mental health specialists trained by psychologists provide the intervention. The training lasted one day and included theoretical background and conversational practice with role-plays. The providers received a manual with all information regarding the intervention.

		preferably a consulting cardiac surgeon performs the operation, alternatively an experienced intern under supervision of a consultant with profound surgical skills, i.e. minimally-invasive access routes. During the postoperive course, the ward physician in consultation with the operating surgeon is responsible for patient care.		start the physiotherapeutic intervention, they should be informed about the treatment rationale in the ERAS concept and peculiarities of the treatment, e.g., early mobilization after extubation.	
**HOW**	The education, and the anesthetic, and postsurgery care is conducted face-to-face.	Every surgical intervention is provided face-to-face.	The intervention is delivered face-to-face individually. Patiens and their relatives can additionally contact the ERAS nurse via telephone, video call, or e-mail.	The intervention is delivered face-to-face individually or, in cases with affiliated persons or relatives of the patient, in small groups. The preoperative session could also be delivered by videocall.	The first three consultations are face-to-face. If needed, there can be telephone calls in between the consultations. The last consultation is planned as a telephone call. All sessions should be done individually. Relatives can participate in the conversations if the patient wants them to.

**WHERE**	The perioperative anesthetic care takes place in the operation room, and the anesthesica care unit.	The preoperative intervention takes place in the outpatient clinic.<br>The intraoperative intervention takes place in the operating room.<br>The postoperative intervention takes place at the surgical ward.	The intervention takes place in different rooms of the hospital. The preliminary talk takes place in a quiet room that provides privacy. The patients are treated in an inpatient setting on a normal ward and in the PACU/ICU/IMC.	The intervention occurs in the hospital on different wards; thus, the locations differ. For the pre-surgery session, any separate room with chairs and maybe a table should be suitable, as long as it provides privacy and a calm atmosphere.<br>The first few postsurgery sessions will occur in the IMC, PACU, or ICU; the later postsurgery sessions will occur in the peripheral ward, the corridor, the staircases, and/or the clinic campus. There is no specific infrastructure needed; the intervention can be adapted according to the patient-centered goals and the surrounding.	The first consultation should take place in a meeting room or some other quiet and private location. The second and third consultations can take place in the patient´s hospital room. If other quiet places are available, that is possible as well. For the last consultation, the patient and the provider need a telephone, and quiet surroundings would be beneficial.
**WHEN, and HOW MUCH**	The anesthesiologic interventions occur pre-surgery for education, and informed consent, intra- and postsurgery in the PACU.<br>Anesthesiologists join the daily ward rounds until discharge and deliver educational information or additional pain management if needed.	Preoperatively, the surgical intervention occurs once for education about the planned surgical procedure and informed consent.<br>The intraoperative surgical intervention occurs is the performance of the surgery itself.<br>During the postoperative course, ward rounds are performed once a day until discharge, ideally as part of the interprofessional round.	Pre-surgery: the patients and their relatives receive training and advice from the ERAS Nurse on the day of the preliminary consultation. This takes about 30 minutes.<br>Postsurgery: During the inpatient process, the patients are visited daily by the ERAS nurse. This individual treatment takes about 1-3 hours per day.<br>The follow-up treatment in the form of telephone contact with the patient takes about 15 minutes, and the follow-up treatment sometimes includes multiple telephone calls.	Pre-surgery session: one educational session of about 45 minutes in length, scheduled approximately two to three weeks before the surgery.<br>Postsurgery sessions: On the day of the surgery, one or two physiotherapy sessions start approximately three hours after extubation, and with a time interval of three hours before the next physiotherapy session. During the next postsurgery day(s), one or two physiotherapy sessions might be necessary, depending on the individual patient. It is also possible that some patients only need a few physiotherapy sessions after the surgery.<br>The single physiotherapy session lasts between five and thirty minutes, subject to the content of the physiotherapy.<br>These sessions describe only the time spent with a physiotherapist. They do not cover the time patients should spend being physically active, managed on their own, and according to their desired goal.	The first consultation takes place in the context of the interdisciplinary patients´ consultation, which is dated two to three weeks before the surgery. After patients have seen the other disciplines involved in the process (sergeant, anesthesiologist, ERAS-Nurse, physiotherapist), the psychosomatic consultation is the last part of this day and takes about 60 Minutes.<br>The second planned consultation takes place one day prior to surgery. The duration of this session may vary from patient to patient. It can take between 10 and 45 minutes.<br>If patients need to contact the psychosomatic expert, they can call them between the first and the second meeting.<br>The third consultation should take place a few days after the surgery. Depending on the patient´s need, we suggest two to three days afterward. Usually, the sessions are around 15 minutes. If needed, there can be more consultations, even on a daily base.<br>The patients receive a final telephone call four to six weeks after the surgery. This takes

					around 15 minutes. Furthermore, the patients can call the contact after they have left the clinic.
**TAILORING**	All interventions in the different professions are individualized depending on the patient´s individual goals and the bio-psycho-social conditions. The individual tailoring of the INCREASE intervention is elaborated, planned, and evaluated by the interprofessional team together with the patients and their relatives. In addition, intraprofessional tailoring for the different professions takes place by adapting the different nursing, physiotherapeutic, and psychosomatic appointments.				

References

1. Engelman DT, Ben Ali W, Williams JB, Perrault LP, Reddy VS, Arora RC, et al. Guidelines for perioperative care in cardiac surgery: Enhanced recovery after surgery society recommendations. JAMA Surg. 2019;154(8):755.
2. Society E. Nursing & AHPs ERAS® Society; 2021, available from: https://erassociety.org/specialty/nursing-ahps/.
3. Bernet M, Lipp I, Zürcher S, Fuhrler L, Sommerhalder K. Enhanced Recovery after Surgery (ERAS): Wie erle-ben Patientinnen und Patienten den Einbezug in die Pflege? Eine qualitative Perspektive. Eine qualitative Perspektive QuPuG. 2018;5(1):16-25.
4. Brodersen F. Die ERAS-Nurse: Pflegeexpertise in der Chirurgie. Im OP. 2022;12(05):206-10.
5. Teixeira UF, Goldoni MB, Waechter FL, Sampaio JA, Mendes FF, Fontes PRO. Enhanced Recovery (ERAS) nach Leberoperationen: Vergleichsstudie in einem brasilianischen Tertiärzentrum. ABCD. Arquivos Brasileiros de Cirurgia Digestiva. São Paulo2019 2019.
6. Jeff A, Taylor C. Ward nurses' experience of enhanced recovery after surgery: a grounded theory approach. Gastrointest Nurs. 2014;12(4):23-31.
7. Balfour A, Burch J, Fecher-Jones I, Carter FJ. Exploring the fundamental aspects of the Enhanced Recovery After Surgery nurse's role. Nurs Stand. 2019.
8. Abeles A, Kwasnicki RM, Pettengell C, Murphy J, Darzi A. The relationship between physical activity and post-operative length of hospital stay: A systematic review. Int J Surg. 2017;44:295-302.
9. Brooks NA, Kokorovic A, McGrath JS, Kassouf W, Collins JW, Black PC, et al. Critical analysis of quality of life and cost-effectiveness of enhanced recovery after surgery (ERAS) for patient's undergoing urologic oncology surgery: a systematic review. World J Urol. 2022;40(6):1325-42.
10. Noba L, Rodgers S, Chandler C, Balfour A, Hariharan D, Yip VS. Enhanced recovery after surgery (ERAS) reduces hospital costs and improve clinical outcomes in liver surgery: A systematic review and meta-analysis. J Gastrointest Surg. 2020;24(4):918-32.
11. Hickmann E, Richter P, Schlieter H. All together now - patient engagement, patient empowerment, and associated terms in personal healthcare. BMC Health Serv Res. 2022;22(1):1116.
12. Hulzebos EHJ, Smit Y, Helders PPJM, van Meeteren NLU. Preoperative physical therapy for elective cardiac surgery patients. Cochrane Database Syst Rev. 2012;11:CD010118.
13. Zheng Y-T, Zhang J-X. Preoperative exercise and recovery after cardiac surgery: a meta-analysis. BMC Cardiovasc Disord. 2020;20(1):2.
14. Gardner T, Refshauge K, McAuley J, Hübscher M, Goodall S, Smith L. Combined education and patient-led goal setting intervention reduced chronic low back pain disability and intensity at 12 months: a randomised controlled trial. Br J Sports Med. 2019;53(22):1424-31.
15. Ng SX, Wang W, Shen Q, Toh ZA, He HG. The effectiveness of preoperative education interventions on improving perioperative outcomes of adult patients undergoing cardiac surgery: a systematic review and meta-analysis. Eur J Cardiovasc Nurs. 2022;21(6):521-36.
16. Burgess LC, Arundel J, Wainwright TW. The effect of preoperative education on psychological, clinical and economic outcomes in elective spinal surgery: A systematic review. Healthcare (Basel). 2019;7(1):48.
17. Johansson K, Salanterä S, Katajisto J. Empowering orthopaedic patients through preadmission education: results from a clinical study. Patient Educ Couns. 2007;66(1):84-91.
18. Taylor NF, Harding KE, Dennett AM, Febrey S, Warmoth K, Hall AJ, et al. Behaviour change interventions to increase physical activity in hospitalised patients: a systematic review, meta-analysis and meta-regression. Age Ageing. 2022;51(1).
19. Hoyer EH, Friedman M, Lavezza A, Wagner-Kosmakos K, Lewis-Cherry R, Skolnik JL, et al. Promoting mobility and reducing length of stay in hospitalized general medicine patients: A quality-improvement project. J Hosp Med. 2016;11(5):341-7.

20. Yuri Y, Takabatake S, Nishikawa T, Oka M, Fujiwara T. The effects of a life goal-setting technique in a preventive care program for frail community-dwelling older people: a cluster nonrandomized controlled trial. BMC Geriatr. 2016;16(1).
21. Brinkman C, Baez SE, Genoese F, Hoch JM. Use of goal setting to enhance self-efficacy after sports-related injury: A critically appraised topic. J Sport Rehabil. 2020;29(4):498-502.
22. Butāne L, Spilva-Ekerte L, Skride A, Šmite D. Individually tailored remote physiotherapy program improves participation and autonomy in activities of everyday life along with exercise capacity, self-efficacy, and low-moderate physical activity in patients with pulmonary arterial hypertension: A randomized controlled study. Medicina (Kaunas). 2022;58(5):662.
23. Kanejima Y, Shimogai T, Kitamura M, Ishihara K, Izawa KP. Effect of early mobilization on physical function in patients after cardiac surgery: A systematic review and meta-analysis. Int J Environ Res Public Health. 2020;17(19):7091.
24. Khandhar SJ, Schatz CL, Collins DT, Graling PR, Rosner CM, Mahajan AK, et al. Thoracic enhanced recovery with ambulation after surgery: a 6-year experience. Eur J Cardiothorac Surg. 2018;53(6):1192-8.
25. Mayor MA, Khandhar SJ, Chandy J, Fernando HC. Implementing a thoracic enhanced recovery with ambulation after surgery program: key aspects and challenges. J Thorac Dis. 2018;10(Suppl 32):S3809-S14.
26. Ramos Dos Santos PM, Aquaroni Ricci N, Aparecida Bordignon Suster É, de Moraes Paisani D, Dias Chiavegato L. Effects of early mobilisation in patients after cardiac surgery: a systematic review. Physiotherapy. 2017;103(1):1-12.
27. Koenders N, van Oorsouw R, Seeger JPH, Nijhuis - van der Sanden MWG, van de Glind I, Hoogeboom TJ. "I'm not going to walk, just for the sake of walking…": a qualitative, phenomenological study on physical activity during hospital stay. Disabil Rehabil. 2020;42(1):78-85.
28. Morton L, Cogan N, Kornfält S, Porter Z, Georgiadis E. Baring all: The impact of the hospital gown on patient well-being. Br J Health Psychol. 2020;25(3):452-73.
29. Oliver D. David Oliver: Fighting pyjama paralysis in hospital wards. BMJ. 2017:j2096.
30. Peate I. Putting an end to pyjama paralysis: the benefits. Br J Nurs. 2018;27(9):471.
31. Kohlmann S, Rimington H, Weinman J. Profiling illness perceptions to identify patients at-risk for decline in health status after heart valve replacement. J Psychosom Res. 2012;72(6):427-33.
32. Kohlmann S, Kilbert MS, Ziegler K, Schulz K-H. Supportive care needs in patients with cardiovascular disorders. Patient Educ Couns. 2013;91(3):378-84.
33. Rief W, Shedden-Mora MC, Laferton JAC, Auer C, Petrie KJ, Salzmann S, et al. Preoperative optimization of patient expectations improves long-term outcome in heart surgery patients: results of the randomized controlled PSY-HEART trial. BMC Med. 2017;15(1).
34. Kubitz JC, Schulte-Uentrop L, Zoellner C, Lemke M, Messner-Schmitt A, Kalbacher D, et al. Establishment of an enhanced recovery after surgery protocol in minimally invasive heart valve surgery. PLoS One. 2020;15(4):e0231378.
35. Laferton JAC, Shedden Mora M, Auer CJ, Moosdorf R, Rief W. Enhancing the efficacy of heart surgery by optimizing patients' preoperative expectations: Study protocol of a randomized controlled trial. Am Heart J. 2013;165(1):1-7.
36. Salzmann S, Laferton J, Auer C, Shedden-Mora M, Wambach K, Rief W. Patientenerwartungen optimieren: Beschreibung einer präoperativen Kurzintervention am Beispiel von Patienten vor einer Bypass-Operation. Verhaltenstherapie. 2018;28(3):157-65.
37. Miller WR, Rollnick S. Motivational interviewing. 3. Auflage des Standardwerks in Deutsch ed. Freiburg im Breisgau: Lambertus; 2015.
