# Supplementary material for: An implementation manual for an interprofessional enhanced recovery after surgery protocol in cardiac surgery following international established frameworks
Source: Front Cardiovasc Med. 2024 Jul 22;11:1392881. doi: 10.3389/fcvm.2024.1392881 (PMC11298346; doi:10.3389/fcvm.2024.1392881)
Supplement: Supplementary file 2 [file Datasheet1.pdf]

Consolidated Framework for Implementation Research (CFIR) for the Implementation of ERAS as applied in INCREASE

I Innovation Domain

Our ERAS program as applied in the INCREASE study is a multimodal, complex intervention involving heart surgery, anesthetics, nursing, physiotherapy, and psychosomatics following enhanced recovery after surgery (ERAS) principles with pre-, intra-, and postoperative interventions. A description of the innovation using the TIDieR checklist can be found in Supplemental Material 2.

A. Innovation Source

The innovation was developed based on the ERAS guidelines. ERAS is a multimodal, interprofessional approach to improve patient recovery after surgery and includes pre-, intra- and, postoperative management (1). In this context, “Fast Track” or “Rapid Recovery” are used interchangeably with ERAS. ERAS was initially developed in the 1990s for abdominal and bowel surgery to reduce the physical and psychological burden of surgery and related procedures (2). ERAS programs are also increasingly used in cardiac surgery. After hospitation in the Hospital Sancta Maggiore, São Paulo, Brazil, where ERAS was already incorporated in heart valve surgery, the preceding version of the INCREASE trial was developed and implemented in the University Heart and Vascular Center of the University Medical Center Hamburg-Eppendorf (UKE).

B. Innovation Evidence-Base

The positive effects of ERAS programs have been proven in numerous studies. ERAS led to a lower rate of postoperative atrial fibrillation, a shorter intubation time or an earlier extubation time, a shorter length of stay in intensive care units, and a reduction in the overall length of stay compared to conventional procedures in cardiac surgery. In addition, ERAS may decrease pain intensity, reduce opioid use, increase early postoperative mobility, accelerate oral nutrition build-up, and reduce costs (3, 4). A study proved that a standardized treatment path in cardiac surgery leads to shorter stays in the intensive care unit and faster discharge without compromising patient safety. Moreover, postoperative complications and the length of stay in the hospital could be reduced cost-effectively through pre-, intra-, and postoperative measures (5). The predecessor of the INCREASE study could also be evaluated for its effectiveness. In a retrospective analysis, the first n = 101 patients from the program were compared with a historical cohort before the program was introduced (n = 111). No significant differences between intra- or perioperative complications and mortality could be found in either group. Transfusion was required in 11.9 % of patients in the ERAS group versus 18.9 % in the control group (p = 0.158). There was no difference in postoperative complications between the two

comparison groups. Reintubation was necessary in an insignificant number of control group patients. Approximately 20 % of patients developed atrial fibrillation in the postoperative course. There was no difference in the number of readmissions to the ICU. Reoperations in the postoperative course were required in 8.9 % of the ERAS group versus 11.7% in the control group ($p = 0.504$). The permanent implantation of a pacemaker was comparatively similar in both groups and was necessary in 3 % vs. 3.6 % of the patients ($p = 0.797$). There was no difference in the readmission rate from the rehabilitation facility at 30 days (5).

C. Innovation Relative Advantage

The newly developed, standardized procedure based on the guidelines of the ERAS Society was further developed as part of the INCREASE project. The interprofessional team was expanded to include psychosomatics. Psychological support is one of the missing needs expressed by patients with cardiac diseases (6). Interprofessional care can positively influence patient adherence, functional status, length of hospital stay, and other outcomes. In addition, patients rate the quality of care as higher, and patients are more satisfied.

A main component of the standardized procedure was the prehabilitation of the patients and their relatives identified and implemented as part of our ERAS program. The developed interventions and standards are evidence-based, not eminence-based; see also the study by Gebauer et al. (2023) (5). Another goal is expanding and professionalizing the medical-therapeutic professions, for example, establishing an ERAS nurse, a specially trained nursing specialist with a master's degree.

D. Innovation Adaptability

The newly developed, standardized procedure based on the guidelines of the ERAS Society was further developed as part of the INCREASE project. The interprofessional team was expanded to include psychosomatics. Psychological support is one of the missing needs expressed by patients with cardiac diseases (6). Interprofessional care has the potential to positively influence patient adherence, functional status, length of hospital stay and other outcomes. In addition, patients rate the quality of care as higher and patients are more satisfied.

A main component of the standardized procedure was the prehabilitation of the patients and their relatives identified and implemented as part of our ERAS program. The developed interventions and standards are evidence-based, not eminence-based; see also the study by Gebauer et al. (2023) (5). Another goal is expanding and professionalizing the medical-therapeutic professions, for example, establishing an ERAS nurse, a specially trained nursing specialist with a master's degree.

Due to the two study locations, the developed concept was transferred from the UKE to the University Hospital Augsburg (UKA) in Germany. The compulsory and optional ERAS elements were identified.

Due to the different organizational and structural conditions of the individual facilities, there are minor deviations in the clinics, which seem not to influence the standardized processes or the set goals of the study (see TIDieR checklist). A must-have, for example, is the preparation of a patient diary that the interprofessional team and the patients actively work with. For example, the monitoring station, especially the Post Anesthesia Care Unit (PACU), where the patients are looked after for the first 24 hours after the operation, is nice to have in a clinical setting. An Intensive Care Unit (ICU) or Intermediate Care (IMC) can also be used to monitor patients. Our adaptations have shown that the ERAS program is adaptable and, the identification of mandatory and optional elements simplifies adaptation for other institutions.

E. Innovation Trialability

The innovation can be tested or piloted on a small scale and undone. The implementation effort is very high. It takes a lot of effort and time before the intervention can be tested.

F. Innovation Complexity

A network of admission and rehabilitation facilities, local nursing service providers, and university hospitals forms the pillars of perioperative care for patients and their relatives. Various professions work closely together on an interprofessional basis and design individual therapy with the patient using standardized procedures. The interprofessional team works closely with the patients and their relatives. It comprises surgery, anesthesia, rehabilitation management, physiotherapy, psychosomatics, and nursing. For example, an ERAS nurse, primarily an Advanced Practice Nurse, work as a central case manager and navigator in patient care (7). Multiple steps in a distinct order also characterize the ERAS program, whereas many steps are performed in parallel, see TiDier Checklist.

G. Innovation Design

The innovation is well-designed and packaged, including how it is assembled, bundled, and presented. In order to implement the standardized interventions and measures, additional staff was hired in all professions to care for the patients. A patient diary was designed in close cooperation with the marketing department of the clinical institutions. In addition, a monitoring station was set up in Hamburg and Augsburg, and materials were purchased for it, enabling intensive patient care immediately after the surgical intervention (PACU, IMC).

H. Innovation Cost

The developments and research findings of the ERAS Society have proven that such a standardized procedure has a positive outcome not only on the clinical stay and the rehabilitation of the patients but also on the economic costs on different levels (8, 9). Specifically for ERAS in cardiac surgery, the cost-effectiveness could be demonstrated by a positive impact on clinical and patient outcomes (10).

The complete hospital stay of the ERAS group was significantly shorter compared to the control group (6.1 ± 2.6 vs. 7.7 ± 3.8 days; p = 0.008). This resulted in significant cost savings of €1087.2 per patient (p = 0.003). With regard to the intensive physiotherapy treatment according to the ERAS protocol, the costs for physiotherapy were €94.3 higher compared to the control group (p < 0.001). The total costs of the ERAS group were €11,200.0 ± 3029.6/patient compared to €13,109.8 ± 4527.5/patient of the control group (p = 0.006). Implementing the ERAS protocol resulted in cost savings of €1909.8 per patient (p = 0.006) (10).

II Outer Setting Domain

The German health system is the outer setting, in which the Inner Setting 'hospital' exists. It is a social health insurance system, also called the Bismarck system, financed by income-dependent, graduated contributions. In contrast to national health services, the group of insured persons is restricted to employees instead of universal coverage. Further characteristics of the social health insurance system are the solidarity, subsidiarity, and self-governance principles with the multifaceted administration by public and private providers (11, 12). For detailed information about the German health system, see, for example, Blümel et al. 2020 (11).

A. Critical Incidents

During the implementation of the our ERAS program, the COVID-19 pandemic broke out, disrupting not only the implementation of ERAS but also the healthcare system, structures of society, and personal life (13). This large-scale and unanticipated event drastically decreased elective surgeries (14, 15). Furthermore, the COVID-19 pandemic aggravated the already existing shortage of healthcare workers (16, 17) and shortage of intensive care unit capacities, (18, 19) but they are crucial factors for successful implementation and maintenance of ERAS.

B. Local Attitudes

Germany´s perceptions, attitudes, and beliefs about health topics have changed. Patients are increasingly perceived as active and participatory persons interacting at eye level with the healthcare worker, seeking health information, and making informed decisions about their health (20, 21). This self-responsible role of patients is in line with the ERAS program, calling for patients with high levels of self-efficacy to take an active part in the care process. In addition, several national awareness campaigns like the National Physical Activity Recommendations (22) and the National Action Plan Health Literacy (23) have contributed to shaping sociocultural values and beliefs about health-related topics in German society. Thus, our ERAS program can pick up the thread with the modern concept of care in patients with minimally invasive valve surgery.

C. Local Conditions

Several characteristics of the German health care system support the implementation and delivery of the ERAS program as applied in the INCREASE study, like the coverage of the costs by social health insurance (12). On the other hand, the sectorization in the health care system with primary, secondary, and tertiary care is to be overcome by a different type of health care (24). The demographic changes in society (25) also contribute to the drive for change for implementing ERAS with its focus on an active patient role: the shortage of healthcare workers (16, 17), especially in rural areas of Germany (26), requires more engagement of patients and their affiliated and relatives. Moreover, people are retiring at a high age, with some willing or being forced to work even after retirement age (27). Hence, most of the patients in the ERAS program are still working and striving to return to work as one of the rehabilitation goals.

D. Partnerships & Connections

The Inner Setting of the ERAS program, the tertiary care hospital, is networked with different healthcare providers in the inpatient and outpatient sectors, like cardiology practices, communal hospitals, and rehabilitation facilities. Furthermore, the German Heart Foundation (Deutsche Herzstiftung) and a statutory health insurer accompanied the development and implementation of our ERAS program, using the perspectives, ideas, and solutions of an intersectoral network (28).

E. Policies & Laws

Internationally, the existing ERAS guidelines in general (29) and the ERAS guidelines for perioperative care in cardiac surgery in particular (30) support the implementation and delivery of the ERAS program. On the national level, several health policy reforms in the last two decades have changed the provision and financing of hospital care, facilitating but also necessitating the implementation of ERAS (11). Among these policy reforms, the introduction of German diagnosis-related groups (G-DRG) substantially impacted the provision of inpatient care. This classification system is regularly adapted as a result of changes in the health care system, e.g., medical innovations (31). One crucial facilitator for the development and implementation of our ERAS program was the funding provided by the Innovationsausschuss in the Gemeinsamer Bundesausschuss with resources of the Innovationsfonds for promoting new forms of care (Section 92a (1) SGB V). This instrument strives for the successful development and implementation of evidence-based changes in the healthcare for patients within the social health insurance system (32).

F. Financing

The Innovationsausschuss in the Gemeinsamer Bundesausschuss funded the initial development, implementation, and evaluation of the INCREASE study (funding code: 01NVF19028) (7). With a positive evaluation and a recommendation from the Gemeinsamer Bundesausschuss after project

completion, ERAS as applied in INCREASE has the chance to become part of the routine care with coverage of the costs for each patient by their health insurance within the German health system with insurance obligation (11).

G. External Pressure

1. Societal Pressure

Health is a global megatrend. The definition of “health” has broadened, and patients have developed a different awareness of their health. With digitalization and the internet era, patients are increasingly informed, changing the role and the expectations of patients. Shared decision-making, patient empowerment, and health literacy have been encouraged in health promotion efforts, leading to changes in care and care models (33, 34).

2. Market Pressure

Inpatient reimbursement in Germany is based on Diagnosis-Related Groups, which are constantly refined and adapted for the German healthcare system (German Diagnosis-Related Groups G-DRG). It is a classification system for a flat-rate billing procedure assigning patients based on medical data as hospital cases to case groups. Implementing the G-DRG system has introduced competition among hospitals based on hospital cases and length of stay (11, 12). ERAS programs are associated with shorter lengths of stay (10) and interest to the reimbursement system. Analysis from an ERAS program in colorectal resections demonstrated that implementation of ERAS within the G-DRG system is feasible at the cost-covering level (35).

3. Performance-Measurement Pressure

ERAS programs, officially certified by the ERAS Society, participate in the international ERAS Society Interactive Audit System (EIAS) database. This database allows for driving the implementation and delivery of the program and for benchmarking across the participating institutions and their ERAS programs. Nonetheless, this benchmarking is only accessible for certified programs, associated with specifications and costs, and thus might not be suitable for all ERAS programs (36).

III Inner Setting Domain

A. Structural Characteristics

The ERAS program can be implemented in maximum care hospitals with cardiac surgery departments. In our case, these are university hospitals. In Germany, there are 36 university hospitals at the moment. They must all be maximum-care facilities and are part of the respective university (37).

1. Physical Infrastructure

Some characteristics regarding the physical infrastructure support the functional performance of the Inner Setting. For the preoperative patient education, we recommend having a quiet room that provides privacy. Furthermore, hospitals that want to implement ERAS need an operation room for cardiac surgery. Preferably, patients would be transferred to a PACU or IMC. ERAS patients require less intensive monitoring than the ICU would offer and usually only stay there for one night. Therefore, ICU and PACU help to relieve ICUs. This is not only an advantage regarding the high cost of an ICU bed but also the generally increasing volume of patients requiring intensive care.

Furthermore, establishing an ICU or PACU allows hospitals to manage rare resources efficiently and to provide adequate care. If there is no ICU or PACU, patients can be transferred to an ICU after surgery. Moreover, hospitals need a cardio-surgical ward to treat patients after leaving PACU or IMC (38).

2. Information Technology Infrastructure

For the functional performance of ICNREASE, technological systems for telecommunication (access to emails and telephone), electronic documentation (i.e., electronic patient files), and data storage, management, reporting, and analysis are needed. A uniform system promotes coordination between specialists and leads to higher-quality health management of patients (39).

3. Work Infrastructure

The arrangement of tasks, responsibilities, and resources within and between the various teams in a university hospital setting and the delegation of tasks among supervisors and subordinates plays a crucial role in the Inner Setting work. Therefore, a general organigram of the hospital is mandatory. In general, the clinic should have a functioning infrastructure. An arrangement of schedules, shifts, and on-call duties should be available. Moreover, the order of work tasks and procedures should be clear, and managing of workloads is helpful. Standard operating procedures (SOPs) are supportive tools that should be available for any profession and their tasks regarding the ERAS program. They guide professionals involved in ERAS to perform their tasks and promote processes to run automatically. Furthermore, a direct and transparent way of communication is crucial.

B. Relational Connections

The Inner Setting boundaries (e.g., structural and professional) consist of high-quality formal and informal relationships, networks, and teams. Relational connections are a fundamental part of the interprofessional ERAS program. Formal and informal connections have been developed within and across the university hospital as the Inner Setting. These relationships evolve to build a sense of ‘teamwork’ or ‘community’ that may contribute positively to implementation outcomes (40).

Informational relationships within the university hospital include, for example, discussing patient cases between different medical colleagues and interning in other hospitals. Moreover, formal relations are, for example, regular exchanges between hospitals, rehabilitation clinics, and referring clinics or doctors. The HAM-NET Symposium, a local symposium dedicated to health services research in Hamburg, Germany, is an example of formal relations where connections between stakeholders are strengthened (41).

C. Communications

High-quality formal and informal information-sharing practices within and across the university hospital exist. An example of a high-quality formal communication tool is the electronic patient file. Furthermore, it is a central element of the communication for ERAS. All information about a patient is available here, and all professionals working with the patient can get or share information. Moreover, daily interprofessional rounds are a central part of formal communication. Besides that, regular meetings between all the different stakeholders are necessary. Nevertheless, examples of important ways of communication are any patient-related conversations or transfer of information between colleges outside of meetings.

D. Culture

There are shared values, beliefs, and norms across the university hospital. The first two university hospitals in Germany that implemented the ERAS program as applied in INCREASE have set general guidelines and principles. For example, at the UKE, several principles are subsumed under the headline "knowledge, research, and healing". (https://www.uke.de/allgemein/ueber-uns/leitbild/index.html) The principles of research ethics, according to the World Medical Association Declaration of Helsinki (2013) (42), should be followed. They guide the protection of human participants in medical research. The guiding principles include protecting patient health; knowledge cannot trample rights, and following local regulatory norms. Furthermore, the ethics of Beauchamp and Childress (2019) (43) are principles to be respected: autonomy, non-maleficence, beneficence, and justice.

1. Human Equality-Centeredness

In Germany, several laws ensure that human equality-centeredness is lived. Examples are the "Basic Law" (Grundgesetz). The central core element of the Basic Law is that "human dignity is inviolable: it must be respected and protected" (Article 1, Grundgesetz). Besides that, the "General Equal Treatment Act" (Allgemeines Gleichbehandlungsgesetz – AGG) protects people who are discriminated against on racial grounds or because of ethnic origin, gender, religion or belief, disability, age or sexual identity. Every practicing physician must swear to practice according to *The Hippocratic Oath formulated in Geneva* by the World Medical Association (44). Since 2017, an updated version has emphasized the

patient´s autonomy of the patient more strongly than the original version from 1948. Moreover, the ethical and hospital principles mentioned protect the equal value and worth inherent in all human beings. At the UKE, for example, an Equal Opportunity Unit is an integral part of the institution to ensure equal opportunities and equality for all people in the UKE.

2. Recipient-Centeredness

The German laws addressing recipient-centeredness, needs, and welfare are the social legislation and their code of law (Sozialgesetzbuch - SGB). It contains provisions for statutory health insurance, statutory accident insurance, statutory pension insurance, social long-term care insurance, employment promotion (in part), social assistance, and essential benefits for job-seekers. SGB V states that its task is to maintain, restore, or improve the health of the insured, as well as to educate and advise the insured and to work towards a healthy lifestyle.

3. Deliverer-Centeredness

There are laws in Germany that ensure deliverer-centeredness. An example is the "Occupational Health and Safety Act" (Arbeitsschutzgesetz – ArbSchG). It is the essential occupational health and safety obligations of the employer, the obligations and rights of employees, and the monitoring of occupational health and safety following this Act for all areas of activity. Furthermore, in Germany exists the "Statutory Vacation Entitlement" (Bundesurlaubsgesetz – BurlG § 3). All employees in Germany are entitled to paid annual leave.

Additionally, university hospitals often offer occupational health promotion. It usually includes, for instance, free exercise and relaxation offers, discounts on gym memberships, health information events, and more. All these aspects are addressing the needs and welfare of deliverers.

4. Learning-Centeredness

There are shared values, beliefs, and norms around psychological safety, continual improvement, and the use of data to inform practice. Ideally, continuous learning occurs throughout the Inner Setting with visible evidence of engaged process, use of data to inform change, and the necessary relational environment (40, 45, 46). Evidence-based training and study of all professions support learning-centeredness in university hospitals. Furthermore, educational leave, remuneration, and potential analysis promote learning-centeredness.

E. Tension for Change

There is a massive tension for change in the healthcare sector. In general, we face the challenge of more and more patients needing treatment or surgery, and fewer people working in the healthcare sector can provide those treatments (47). Furthermore, the current situation in surgery is

characterized by various deficits like inhomogeneous surgical care quality due to variations in surgical training and differences in quality control (48, 49).

F. Compatibility

The ERAS program was already slightly modified when implemented in a second hospital. It was developed and adapted to the current problem, i.e., pandemic, personal resources, and patient needs. There are some mandatory elements, but also many optional elements. This flexibility makes ERAS compatible for many university hospitals and other maximum-care hospitals. ERAS fits with existing workflows, systems, and processes in maximum-care hospitals.

G. Relative Priority

Implementing and delivering ERAS can be cost-saving due to relief of the ICU with its high personnel requirements (1, 10). ERAS empowers patients; e.g., the fast mobilization after surgery enables patients to provide for themselves and take self-responsibility. That, in turn, is one way to adapt to the shortage and lack of specialists in all clinical professions, as patients need less assistance. Furthermore, employee satisfaction in ERAS programs seems to be another argument favoring relative priority (50).

H. Incentive Systems

Tangible and intangible incentives and rewards support the implementation and delivery of the innovation. A tangible incentive for the hospital´s economy is that ERAS might save costs. Reasons for that are, i.e., the IMC instead of ICU stay and the shorter hospital stay of patients in ERAS programs than other patients. The pilot study of the INCREASE trial showed significant cost savings of € 1,087.20 per patient (10). An example of intangible incentives is that every profession, part of ERAS, might benefit from a better and more visible reputation through the interprofessional focus.

Moreover, positive patient feedback is a motivating factor for all professionals involved (51). The INCREASE trial is an excellent example of ERAS benchmarking. Benchmarking helps to continuously improve the ERAS process by learning from the experiences and best practices of others and applying them. ERAS benchmarking is a tool for comparing the performance and effectiveness of ERAS programs across medical facilities and identifying best practices (36). INCREASE is an excellent example because of its robust methods, relevance, innovation, and broad impact.

I. Mission Alignment

Implementing and delivering ERAS aligns with the Inner Setting´s overarching commitment, purpose, and goals.

J. Available Resources

Provision of funding, space, materials, and equipment are essential for the implementation and delivery of the innovation.

1. Funding

Statutory health insurance must cover the costs for counseling and patient education, especially pre-operative in the future. We recommend adding an ERAS nurse to the general list of healthcare personnel. The nurse plays a vital role in implementing, coordinating, and overseeing ERAS protocols. They ensure specialized postoperative care, patient education, protocol adherence, data monitoring, and emotional support to facilitate a smoother and faster patient recovery process. Furthermore, the nurse is the main contact person for patients, their relatives, and everyone involved in the care process.

2. Space

ERAS only requires space that is already standard at a university hospital or a maximum-care hospital. Every university hospital should have a PACU, IMC, or ICU. A physiotherapy room with training equipment like a treadmill or cycle ergometer and a separate and quiet room for preoperative education might be beneficial, but these are not mandatory.

3. Materials & Equipment

The PACU or IMC should have all the gear necessary for patients after heart surgery (52). We highly recommend using a patient journal/ diary with extra information and exercises to prepare for surgery and to guide patients afterward. It is the permanent companion of the patient.

K. Access to Knowledge & Information

Guidance and training must be accessible to implement and deliver ERAS successfully. General information about ERAS can be found at the ERAS Society (https://erassociety.org/). The German Society for Thoracic, Cardiac, and Vascular Surgery (Deutsche Gesellschaft für Thorax-, Herz- und Gefäßchirurgie – DGTHG) provides information about requirements and standards in cardiac surgery in Germany (https://www.dgthg.de/). Furthermore, visiting congresses or lectures might deliver helpful insights into the newest topics. Moreover, this present guideline provides support for implementing ERAS.

IV Individuals Domain

Roles Subdomain

Note: Both natural and legal persons can fill the roles as part of the ERAS program.

Need: *The individual(s) has deficits related to survival, well-being, or personal fulfillment, which will be addressed by the implementation and/or delivery of the innovation.*
Capability: *The individual(s) has interpersonal competence, knowledge, and skills to fulfill the role.*
Opportunity: *The individual(s) has the availability, scope, and power to fulfill the role.*
Motivation: *The individual(s) is committed to fulfilling the role.*
The description and definition of each role can be found in the supplemental material of the updated CFIR (53).

Roles:	Characteristics:			
	**Need**	**Capability**	**Opportunity**	**Motivation**
**A. High-level Leaders:** Medical director/CEO	Cost efficiency clinic management, evidence-based care at minimal cost	Decision on introducing implementation of new methods in own hospital,	Long-term cost reduction, efficient patient care, satisfied employees, satisfied patients, transferability to other areas	Economic benefits, prestigious, offering innovative, evidence-based methods, increase in patient numbers, increasing the popularity of the hospital
Ministry of Health; "Joint Federal Committee" (Gemeinsamer Bundesausschuss), has the power to implement ERAS into the benefits catalog of the social health insurance	Cost-effectiveness, performance of health system, evidence-based care for population	Political power, the decision to introduce the benefits catalog of the social health insurance companies	Long-term cost reduction, efficient patient care, satisfied patients, transferability to other areas	Economic benefits, innovative, evidence-based methods, cutting-edge medicine location Germany
**B. Mid-level Leaders** Directors of referring clinics, heads of the involved departments	Fast and best possible care; high patient satisfaction, optimal personnel planning, cost-efficiency	Staff scheduling, transmission of new methods, employee training	Motivated and well-trained employees, interdisciplinary exchange of experience, patient satisfaction	Optimal staff planning, seamless patient care, employee engagement
**C. Opinion Leaders** Referring clinics, Rehabilitation clinics, "German Heart Foundation" (Deutsche Herzstiftung), all of the other leaders mentioned above; potential opinion leaders to be involved in the future: professional associations, patient representatives of the federal government; can be anyone	Improving the current standard of care, implementation of evidence in clinical practice, learning from other professions, and more appreciation for each profession involved	Information dissemination, education, and motivation of all other people involved	Self-fulfillment, wide dissemination of knowledge, ERAS as standard care, advancement of professions, development of quality standards in care	Achieving the best possible patient care, sharing of own knowledge, increasing attraction of professions
**D. Implementation Facilitators** Professional organizations (e.g., ERAS Society), representatives of the individual disciplines (trained experts; different prerequisites/experience from different professional groups)	Nationwide implementation of developed standard care programs in other institutions	Expert knowledge about the implementation and content of the innovation	Increase in awareness of the importance of each profession involved and interprofessional cooperation, ERAS as a standard	As many people as possible should benefit, improve viewpoints of one's own discipline, sharing knowledge and experience

Roles:	Characteristics:			
	**Need**	**Capability**	**Opportunity**	**Motivation**
**E. Implementation Leads** can be anyone with motivation; interface/main coordinator	Improvement of patient care	Skilled in communication, having a general overview of the structures and processes of the institution	Successful implementation of ERAS	Motivation and enthusiasm for ERAS, want to spread ERAS, to motivate other individuals for the program
**F. Implementation Team Members** One main responsible from each profession, at least a Mid-level leader, and ideally patient representatives → professional realization	Further development of own activity/area of work, workload at a manageable and appropriate level, interprofessional cooperation	Evidence-based expertise, appropriate training, collaborative skills	Learning from exchange with other disciplines, self-realization at work, further development/education	Convinced of ERAS, strengthen its area of expertise, make patient care as effective as possible, perform qualitatively good work
**G. Other Implementation Support** Repetition from High Level: Person with decision-making power, senior managers	Cost efficiency clinic management, evidence-based care at minimal cost	Decision on introducing implementation of new methods in own hospital, knowledge about ERAS	Long-term cost reduction, efficient patient care, satisfied patients and employees, transferability to other areas, employee engagement	Economic benefits, prestigious, offering innovative, evidence-based methods, increase in patient numbers, increasing the popularity of the hospital
**H. Innovation Deliverers** All professionals involved in the treatment of the patients or the administration processes of the clinic	Evidence-based care, fulfilling work requirements, complying with SOPs	Knowledge about ERAS and competencies in the individual steps that are relevant for the profession	Conviction and enthusiasm for the program, gain more knowledge, personal development	Implement ERAS and bring it to the patients
**I. Innovation Recipients** Patients and relatives	Improvement of care, receive the best possible care	Understanding to participate (i.e., language, the time required for the preliminary interview, consent), physical and mental capability	Increase in quality of life, improving health behavior, empowerment, self-efficacy, intrinsic motivation	Active participation in care, faster recovery, and support of the patient

V Implementation Process Domain

This domain describes the relevant activities and strategies used to implement the innovation. In addition to these activities and strategies, some prerequisites, especially for the different professions but also cross-sectoral, can be found in the TIDieR. These prerequisites have to be implemented before the start of the INCREASE program.

A. Teaming

Teaming is the process of joining together and intentionally coordinating and collaborating on interdependent tasks to implement innovation (40). Meetings of the representatives of the individual disciplines for team building should take place consciously. According to our experience of implementing the ERAS program, an important point of time for teaming was the project kick-off, the onboarding new team members, and off-boarding team members. Team meetings, team buildings, regular jour fixes, and exchanges on different levels between different professions were crucial for the teaming process. In the practical care of patients and relatives, the daily visits by the interprofessional team are part of the teaming.

B. Assessing Needs

Assessing the needs of both recipients and deliverers is crucial to guide the implementation process and ensure implementation success and equity (54, 55). When assessing those needs, one gathers information about individuals´ priorities, preferences, and requirements. Perform a literature search about published priorities, preferences, and needs and adapt them to the local conditions. Furthermore, consider quality indicatorsresulting from a structured consensus such as Delphi study methods (56).

1. Innovation Deliverers

Assessing the needs of deliverers is crucial for achieving the 'Quadruple Aim,' which aims to improve the work-life and well-being of clinicians and staff (57). Therefore, collecting information about deliverers´ priorities, preferences, and needs is essential to guide the innovations´ implementation and delivery. We found feedback talks, questionnaires, and interviews to be adequate tools for this assessment. Furthermore, each profession should have the opportunity to set out and address their perspectives, attitudes, beliefs, and their point of view.

2. Innovation Recipients

Assessing recipient needs helps to promote patient-centered care and a culture of patient safety within healthcare delivery (58). This involves gathering information about recipients ‘priorities,

preferences, and needs to guide the innovations implementation and delivery. We assessed recipient needs through feedback talks, questionnaires, and interviews.

C. Assessing Context

Assessing context is fundamental in implementation science (59). Therefore, it is necessary to gather information to identify and evaluate the obstacles and aids to implementing and delivering the innovation. We used the present CFIR framework and the resulting implementation guideline to assess relevant context factors, e.g., financing, health system, or knowledge of deliverer. Moreover, other corporatist committees influenced the genesis, implementation, and review of innovations in the German healthcare system, such as the "Expert Council for the Assessment of Developments in the Health Care System" (formerly the Concerted Action in Health Care) or any commissions such as the Round Table from 2015 2001 or the Rürup Commission from 2002 (60).

D. Planning

The primary goal of planning is to create a plan of action that facilitates effective implementation by developing local capacity for utilizing the innovation, collectively and individually (61). To initiate implementation planning, it is essential to identify roles and responsibilities, outline specific steps and milestones, and establish objectives and metrics for measuring implementation success in advance. Important milestones for our implementation of ERAS were, for example, the setup of PACU and IMC, training for employees, the kick-off, and the first ten treated patients. Moreover, coordinating the plan via project management was essential for the successful implementation. Examples of how we measured the implementation success were the time point of hospital discharge and the length of hospital stay. Additionally, we defined a responsible person for each profession and responsible persons for each department and other organizational structures.

E. Tailoring Strategies

Implementation strategies are selected and customized to address the results of the Needs Assessment and Context Assessment (62). Therefore, we choose and implement strategies to overcome barriers, utilize facilitators, and fit the context. One example of a barrier we faced was the COVID-19 pandemic with its consequences for the healthcare sector. A restriction that affected our ERAS program, was the limitation of visitors and relatives allowed in the hospital. Moreover, fewer surgeries were planned, and there was limited personnel in all areas.

F. Engaging

This construct involves attracting and involving the appropriate individuals, specifically deliverers, and recipients, in implementing and using the innovation. We tried to attract and encourage participation in implementation and innovation.

1. Innovation Deliverers

We aimed to attract and encourage deliverers to serve on the implementation team and to deliver the innovation. To achieve this, we found regular meetings and exchanges essential. Furthermore, it is essential to show the benefits of the ERAS program to the deliverers, such as a more efficient work, positive patient feedback, working in an interprofessional team, and learning from the interprofessional exchange. Moreover, we found it advantageous to include the deliverers in decision-making and enable their active participation.

2. Innovation Recipients

Attracting and encouraging recipients to serve on the implementation team and/or participate in the innovation is also crucial. We made the recipients curious about the INCREASE trial via marketing on several levels (referral clinics, television, homepage, newspaper…). When showing recipients the benefits of participating in the INCREASE trial, we successfully encouraged them to engage. Furthermore, patients were also integrated into the development of the ERAS program, for example, by including their feedback in the process.

G. Doing

Doing involves using quality improvement methods, such as Plan-Do-Study-Act cycles or incremental steps, instead of implementing all components in all planned areas within a specified period (63). It might be helpful to implement modifications in small units to test and cumulatively optimize the delivery of the innovation. Therefore, we recommend testing the implementation of the ERAS program with a pilot phase. After treating the first ten patients, a feedback and adjusting phase might be appropriate. Furthermore, we suggest starting with a few patients per week and then slowly increasing the number of patients, the clinic´s capacity, and the visibility and advertisement for the program.

H. Reflecting & Evaluating

We collected and discussed quantitative and qualitative information about the success of implementation and innovation. We evaluated the first test and the pilot phase and derived adjustments. Furthermore, from the first data collection, we could evaluate several components of ERAS, for example, the preferable time to remove the central venous catheter.

1. Implementation

Here, we want to collect and discuss quantitative and qualitative information about the success of the implementation of ERAS. Measurements of success are, for instance, if a routine process prevails during the complete perioperative program. Furthermore, all required materials must

be available (e.g., equipment for PCAU/ IMC, patient diary), and all involved persons must be trained sufficiently.

2. Innovation

We collected and discussed quantitative and qualitative information about the success of the innovation. That indicates the degree to which the innovation outcomes are achieved. We evaluate the innovation as successful when the hospital discharge could occur within the planned length of stay. The number of complications, such as infections or delirium, is used as a further indicator of the success of the innovation. Moreover, the satisfaction of deliverers and recipients and the health-related quality of life of the recipients are essential measurements.

I. Adapting

The last step of the implementation process is to modify the innovation and/or the Inner Setting for optimal fit and integration into work processes. We already modified the innovation when transferring the INCREASE study from the UKE to the UKA. According to our experience, a monitoring system would be helpful for continuous outcome collection and evaluation if innovation outcomes were achieved, for example, if patients achieved the time spent outside bed. In general, personnel structure, changes in personnel, political changes, and context factors can always lead to the need to adapt to the implementation.

References

1. Ljungqvist O, Scott M, Fearon KC. Enhanced Recovery After Surgery: A Review. JAMA Surg. 2017;152(3):292-8.
2. Kaye AD, Urman RD, Cornett EM, Hart BM, Chami A, Gayle JA, et al. Enhanced recovery pathways in orthopedic surgery. J Anaesthesiol Clin Pharmacol. 2019;35(Suppl 1):S35-s9.
3. Baxter R, Squiers J, Conner W, Kent M, Fann J, Lobdell K, et al. Enhanced Recovery After Surgery: A Narrative Review of its Application in Cardiac Surgery. Ann Thorac Surg. 2020;109(6):1937-44.
4. Kamal YA, Hassanein A. Do perioperative protocols of enhanced recovery after cardiac surgery improve postoperative outcome? Interact Cardiovasc Thorac Surg. 2020;30(5):706-10.
5. Gebauer A, Konertz J, Petersen J, Brickwedel J, Köster D, Schulte-Uentrop L, et al. The impact of a standardized Enhanced Recovery After Surgery (ERAS) protocol in patients undergoing minimally invasive heart valve surgery. PLOS ONE. 2023;18(3):e0283652.
6. Kohlmann S, Kilbert MS, Ziegler K, Schulz KH. Supportive care needs in patients with cardiovascular disorders. Patient Educ Couns. 2013;91(3):378-84.
7. Klotz SGR, Ketels G, Behrendt CA, König HH, Kohlmann S, Löwe B, et al. Interdisciplinary and cross-sectoral perioperative care model in cardiac surgery: implementation in the setting of minimally invasive heart valve surgery (INCREASE)-study protocol for a randomized controlled trial. Trials. 2022;23(1):528.
8. Noba L, Rodgers S, Chandler C, Balfour A, Hariharan D, Yip VS. Enhanced Recovery After Surgery (ERAS) Reduces Hospital Costs and Improve Clinical Outcomes in Liver Surgery: a Systematic Review and Meta-Analysis. J Gastrointest Surg. 2020;24(4):918-32.
9. Noba L, Rodgers S, Doi L, Chandler C, Hariharan D, Yip V. Costs and clinical benefits of enhanced recovery after surgery (ERAS) in pancreaticoduodenectomy: an updated systematic review and meta-analysis. J Cancer Res Clin Oncol. 2023;149(9):6639-60.
10. Petersen J, Kloth B, Konertz J, Kubitz J, Schulte-Uentrop L, Ketels G, et al. Economic impact of enhanced recovery after surgery protocol in minimally invasive cardiac surgery. BMC Health Serv Res. 2021;21(1):254.
11. Blümel M, Spranger A, Achstetter K, Maresso A, Busse R. Germany: Health System Review. Health Syst Transit. 2020;22(6):1-272.
12. Busse R, Blümel M, Knieps F, Bärnighausen T. Statutory health insurance in Germany: a health system shaped by 135 years of solidarity, self-governance, and competition. Lancet. 2017;390(10097):882-97.
13. Valeras AS. COVID-19: Complexity and the Black Swan. Fam Syst Health. 2020;38(2):221-3.
14. Garofil ND, Bratucu MN, Zurzu M, Paic V, Tigora A, Prunoiu V, et al. Groin Hernia Repair during the COVID-19 Pandemic-A Romanian Nationwide Analysis. Medicina (Kaunas). 2023;59(5).

15. Giuffrida M, Cozzani F, Rossini M, Bonati E, Del Rio P. How COVID-19 pandemic has changed elective surgery: the experience in a general surgery unit at a COVID-hospital. Acta Biomed. 2021;92(5):e2021304.
16. de Vries N, Boone A, Godderis L, Bouman J, Szemik S, Matranga D, et al. The Race to Retain Healthcare Workers: A Systematic Review on Factors that Impact Retention of Nurses and Physicians in Hospitals. Inquiry. 2023;60:469580231159318.
17. De Vries N, Lavreysen O, Boone A, Bouman J, Szemik S, Baranski K, et al. Retaining Healthcare Workers: A Systematic Review of Strategies for Sustaining Power in the Workplace. Healthcare (Basel). 2023;11(13).
18. Dar M, Swamy L, Gavin D, Theodore A. Mechanical-Ventilation Supply and Options for the COVID-19 Pandemic. Leveraging All Available Resources for a Limited Resource in a Crisis. Ann Am Thorac Soc. 2021;18(3):408-16.
19. Knochel K, Schmolke EM, Meier L, Buyx A. Translating theories of justice into a practice model for triage of scarce intensive care resources during a pandemic. Bioethics. 2023.
20. Baumann E, Czerwinski F, Rosset M, Seelig M, Suhr R. [How do people in Germany seek health information? Insights from the first wave of HINTS Germany]. Bundesgesundheitsblatt Gesundheitsforschung Gesundheitsschutz. 2020;63(9):1151-60.
21. Horch K, Wirz J. [People's interest in health information]. Bundesgesundheitsblatt Gesundheitsforschung Gesundheitsschutz. 2005;48(11):1250-5.
22. Füzéki E, Vogt L, Banzer W. [German National Physical Activity Recommendations for Adults and Older Adults: Methods, Database and Rationale]. Gesundheitswesen. 2017;79(S 01):S20-s8.
23. Schaeffer D, Gille S, Hurrelmann K. Implementation of the National Action Plan Health Literacy in Germany-Lessons Learned. Int J Environ Res Public Health. 2020;17(12).
24. Köhler A. [Intersectoral care: sector-linking approaches or a shifting of the sector boundary]. Inn Med (Heidelb). 2022;63(9):923-9.
25. Schmitz MT, Just JM, Weckbecker K, Schmid M, Münster E. [Multimorbidity and its Importance in Future Health Care in Germany: a Secondary Data Analysis Based on 67 Million Health Insurance Policy Holders]. Gesundheitswesen. 2023.
26. Steinhäuser J, Annan N, Roos M, Szecsenyi J, Joos S. [Approaches to reduce shortage of general practitioners in rural areas--results of an online survey of trainee doctors]. Dtsch Med Wochenschr. 2011;136(34-35):1715-9.
27. Hofäcker D, Naumann E. The emerging trend of work beyond retirement age in Germany. Increasing social inequality? Z Gerontol Geriatr. 2015;48(5):473-9.

28. Bevc CA, Retrum JH, Varda DM. New perspectives on the "silo effect": initial comparisons of network structures across public health collaboratives. Am J Public Health. 2015;105 Suppl 2(Suppl 2):S230-5.
29. Ljungqvist O, Francis NK, Urman RD. Enhanced recovery after surgery. A complete guide to optimizing outcomes. Cham: Springer Nature Switzerland; 2020.
30. Engelman DT, Ben Ali W, Williams JB, Perrault LP, Reddy VS, Arora RC, et al. Guidelines for Perioperative Care in Cardiac Surgery: Enhanced Recovery After Surgery Society Recommendations. JAMA Surgery. 2019;154(8):755-66.
31. Franz D, Wenke A, Roeder N. [Depiction of cardiovascular surgery in the current modified German DRG system 2021]. Z Herz Thorax Gefasschir. 2021;35(2):83-96.
32. Hecken J. [Where should the innovation fund develop after stabilisation?]. Gesundheitsökonomie & Qualitätsmanagement. 2023;28(03):128-32.
33. Deml MJ, Jungo KT, Maessen M, Martani A, Ulyte A. Megatrends in Healthcare: Review for the Swiss National Science Foundation's National Research Programme 74 (NRP74) "Smarter Health Care". Public Health Rev. 2022;43:1604434.
34. Hartzband P, Groopman J. Untangling the Web--patients, doctors, and the Internet. N Engl J Med. 2010;362(12):1063-6.
35. Koch F, Green M, Dietrich M, Moikow L, Schmidt M, Ristig M, et al. The usefulness of Enhanced Recovery After Surgery concepts for colorectal resections: an economic analysis under DRG conditions. Langenbecks Arch Surg. 2022;407(7):2981-6.
36. Currie A, Soop M, Demartines N, Fearon K, Kennedy R, Ljungqvist O. Enhanced Recovery After Surgery Interactive Audit System: 10 Years' Experience with an International Web-Based Clinical and Research Perioperative Care Database. Clin Colon Rectal Surg. 2019;32(1):75-81.
37. Deutscher Bundestag. Begriff, Rechtsformen und Finanzierung der Universitätskliniken in Deutschland 2009 [Available from: https://www.bundestag.de/resource/blob/411984/8e841d94363c58f662a320e9fde782d6/WD-9-087-09-pdf-data.pdf.
38. Gummert J, Beckmann A, Bauer A, Heinemann M, Markewitz A, Falk V, et al. Basis-Anforderungen einer Fachabteilung für Herzchirurgie. Thorac Cardiovasc Surg. 2022;70(6):452-7.
39. Yuan S, Wang F, Li X, Jia M, Tian M. Facilitators and barriers to implement the family doctor contracting services in China: findings from a qualitative study. BMJ Open. 2019;9(10):e032444.
40. Edmondson AC. Teaming: How organizations learn, innovate, and compete in the knowledge economy: Jossey-Bass; 2012.

41. Bremer D, Busch S, Liedtke F, Löwe B, Meusch A, Scherer M, et al., editors. HAM-NET – Weiterentwicklung und Verstetigung des Hamburger Netzwerks für Versorgungsforschung. 19 Deutscher Kongress für Versorgungsforschung; 2020; digital: German Medical Science GMS Publishing House.
42. World Medical Association. World Medical Association Declaration of Helsinki: ethical principles for medical research involving human subjects. Jama. 2013;310(20):2191-4.
43. Beauchamp TL, Childress JF. Principles of biomedical ethics. 8 ed. London, England: Oxford University Press; 2019.
44. World Medical Association. Declaration of Geneva 2017 [Available from: https://www.wma.net/policies-post/wma-declaration-of-geneva/.
45. Lapré MA, Nembhard IM. Inside the Organizational Learning Curve: Understanding the Organizational Learning Process. Foundations and Trends in
Technology, Information and Operations Management. 2010;4(1):1-103.
46. Miake-Lye IM, Delevan DM, Ganz DA, Mittman BS, Finley EP. Unpacking organizational readiness for change: an updated systematic review and content analysis of assessments. BMC Health Serv Res. 2020;20(1):106.
47. Wasem J, Blase N. Die Personalentwicklung im Krankenhaus seit 2000. In: Klauber J, Wasem J, Beivers A, Mostert C, editors. Krankenhaus-Report 2023: Schwerpunkt: Personal. Berlin, Heidelberg: Springer Berlin Heidelberg; 2023. p. 3-18.
48. Axt S, Johannink J, Storz P, Mees ST, Röth AA, Kirschniak A. [Surgical Training in Germany: Desire and Reality]. Zentralbl Chir. 2016;141(3):290-6.
49. Rosato L, Lavorini E, Balzi D, Mondini G, Panier Suffat L. Morbidity and mortality analysis in general surgery operations. Is there any room for improvement? Minerva Surg. 2022;77(3):229-36.
50. Angus M, Jackson K, Smurthwaite G, Carrasco R, Mohammad S, Verma R, et al. The implementation of enhanced recovery after surgery (ERAS) in complex spinal surgery. J Spine Surg. 2019;5(1):116-23.
51. Clifford C, Girdauskas E, Klotz SGR, Kurz S, Löwe B, Kohlmann S. Patient-centered evaluation of an expectation-focused intervention for patients undergoing heart valve surgery: A qualitative study. 2024.
52. Simpson JC, Moonesinghe SR. Introduction to the postanaesthetic care unit. Perioper Med (Lond). 2013;2(1):5.
53. Damschroder LJ, Reardon CM, Widerquist MAO, Lowery J. The updated Consolidated Framework for Implementation Research based on user feedback. Implement Sci. 2022;17(1):75.

54. Ashok M, Hung D, Rojas-Smith L, Halpern MT, Harrison M. Framework for Research on Implementation of Process Redesigns. Qual Manag Health Care. 2018;27(1):17-23.
55. Dy SM, Ashok M, Wines RC, Rojas Smith L. A framework to guide implementation research for care transitions interventions. J Healthc Qual. 2015;37(1):41-54.
56. Klotz SGR, Begerow A, Girdauskas E. Development of a Core Outcome Set for Enhanced Recovery After Surgery in Minimally Invasive Cardiac Surgery. Results of an Interprofessional Delphi Consensus Study. 2024.
57. Bodenheimer T, Sinsky C. From triple to quadruple aim: care of the patient requires care of the provider. Ann Fam Med. 2014;12(6):573-6.
58. Nieva VF, Sorra J. Safety culture assessment: a tool for improving patient safety in healthcare organizations. Qual Saf Health Care. 2003;12 Suppl 2(Suppl 2):ii17-23.
59. Nilsen P, Bernhardsson S. Context matters in implementation science: a scoping review of determinant frameworks that describe contextual determinants for implementation outcomes. BMC Health Serv Res. 2019;19(1):189.
60. Hower K, Pförtner T-K, Pfaff H, Wensing M, Ansmann L. Innovationen im Gesundheitswesen. In: Blättel-Mink B, Schulz-Schaeffer I, Windeler A, editors. Handbuch Innovationsforschung: Sozialwissenschaftliche Perspektiven. Wiesbaden: Springer Fachmedien Wiesbaden; 2021. p. 629-48.
61. Mendel P, Meredith LS, Schoenbaum M, Sherbourne CD, Wells KB. Interventions in organizational and community context: a framework for building evidence on dissemination and implementation in health services research. Adm Policy Ment Health. 2008;35(1-2):21-37.
62. Powell BJ, Beidas RS, Lewis CC, Aarons GA, McMillen JC, Proctor EK, et al. Methods to Improve the Selection and Tailoring of Implementation Strategies. J Behav Health Serv Res. 2017;44(2):177-94.
63. Taylor MJ, McNicholas C, Nicolay C, Darzi A, Bell D, Reed JE. Systematic review of the application of the plan-do-study-act method to improve quality in healthcare. BMJ Qual Saf. 2014;23(4):290-8.
